# RNA:DNA hybrids are a novel molecular pattern sensed by TLR9

**DOI:** 10.1002/embj.201386117

**Published:** 2014-02-21

**Authors:** Rachel E Rigby, Lauren M Webb, Karen J Mackenzie, Yue Li, Andrea Leitch, Martin A M Reijns, Rachel J Lundie, Ailsa Revuelta, Donald J Davidson, Sandra Diebold, Yorgo Modis, Andrew S MacDonald, Andrew P Jackson

**Affiliations:** 1 MRC Human Genetics Unit, MRC IGMM, University of EdinburghEdinburgh, UK; 2 Institute of Immunology and Infection Research, University of EdinburghEdinburgh, UK; 3 Department of Molecular Biophysics and Biochemistry, Yale UniversityNew Haven, CT, USA; 4 MRC Centre for Inflammation Research, Queen's Medical Research Institute, The University of EdinburghEdinburgh, UK; 5 Division of Immunology, Infection and Inflammatory Disease, King's College LondonLondon, UK; † MRC Human Immunology Unit, Radcliffe Department of Medicine, MRC WIMM, University of OxfordOxford, UK; ‡ Manchester Collaborative Centre for Inflammation Research, University of ManchesterManchester, UK

**Keywords:** innate immune signalling, pathogen-associated molecular pattern, RNA:DNA hybrid, TLR9

## Abstract

The sensing of nucleic acids by receptors of the innate immune system is a key component of antimicrobial immunity. RNA:DNA hybrids, as essential intracellular replication intermediates generated during infection, could therefore represent a class of previously uncharacterised pathogen-associated molecular patterns sensed by pattern recognition receptors. Here we establish that RNA:DNA hybrids containing viral-derived sequences efficiently induce pro-inflammatory cytokine and antiviral type I interferon production in dendritic cells. We demonstrate that MyD88-dependent signalling is essential for this cytokine response and identify TLR9 as a specific sensor of RNA:DNA hybrids. Hybrids therefore represent a novel molecular pattern sensed by the innate immune system and so could play an important role in host response to viruses and the pathogenesis of autoimmune disease.

See also: **SB Jensen & SR Paludan** (March 2014)

## Introduction

The innate immune system is a key component of host response to infection. It senses molecular patterns associated with pathogens and “danger” using a repertoire of germline-encoded pattern recognition receptors (PRRs) that permit detection of conserved microbial molecular motifs (Matzinger, 1994; Janeway & Medzhitov, 2002). Nucleic acids, as indispensable components of all pathogens, represent an important class of PRR ligand (Barbalat *et al*, 2011). Several families of nucleic acid-sensing PRRs have been characterised, including the membrane-associated Toll-like receptor (TLR) and cytosolic RNA-sensing RIG-I-like receptor (RLR) families (Desmet & Ishii, 2012).

Five TLRs sense nucleic acids: TLR3 (double-stranded RNA, dsRNA), TLR7/8 (single-stranded RNA, ssRNA), TLR9 (bacterial DNA) and TLR13 (23S rRNA) (Hemmi *et al*, 2000; Alexopoulou *et al*, 2001; Diebold *et al*, 2004; Heil *et al*, 2004; Oldenburg *et al*, 2012). These TLRs localise to intracellular membranes, relocating from the ER to endolysosomes via UNC93B1-mediated trafficking (Latz *et al*, 2004; Kim *et al*, 2008), where they undergo proteolytic cleavage to generate a functional receptor that interacts with its nucleic acid ligand (Ewald *et al*, 2008; Park *et al*, 2008). Nucleic acid binding to TLR homodimers recruits the adapter proteins TRIF or MyD88 to trigger NF-κB and/or IRF signalling pathways, inducing pro-inflammatory cytokines and type I interferon (IFN) respectively (Blasius & Beutler, 2010).

Viral genomes (Lund *et al*, 2003; Rehwinkel *et al*, 2010) and their replication intermediates (Lee *et al*, 2007) are detected as non-self nucleic acids in cytosolic and endosomal compartments by PRRs, which induce type I IFN production to establish a potent antiviral response (Samuel, 2001). In some circumstances, the same receptors bind self-nucleic acids, such as those released from damaged cells (Pisetsky & Fairhurst, 2007). This can occur when self-nucleic acids in complex with human cationic host defence peptides such as LL-37 are internalised by antigen presenting cells (APCs) which potently activate PRR signalling cascades and type I IFN production (Lande *et al*, 2007; Ganguly *et al*, 2009). An inflammatory response in the absence of infection also occurs in the childhood onset single gene disorder Aicardi-Goutières syndrome (AGS)(Crow & Livingston, 2008). This genetic mimic of viral infection is caused by mutations in genes encoding four nucleic acid-metabolising enzymes, including Ribonuclease (RNase) H2 (Crow *et al*, 2006b). As RNase H enzymes hydrolyse the RNA strand of RNA:DNA heteroduplexes (Stein & Hausen, 1969), RNA:DNA hybrids are thought to accumulate in RNase H2-deficient AGS patient cells and induce type I IFN through PRR activation (Alarcon-Riquelme, 2006). Physiological sensing of RNA:DNA hybrids may also be immunologically advantageous, as many major pathogenic viruses (including HIV, CMV, EBV and Hepatitis B) generate RNA:DNA hybrid structures during their replication within an infected cell (Summers & Mason, 1982; Prichard *et al*, 1998; Rennekamp & Lieberman, 2011).

Given the essential role of RNA:DNA hybrids in retroviral replication and the postulated accumulation of RNA:DNA hybrids in AGS, we hypothesised that RNA:DNA hybrids may represent an additional category of immunostimulatory nucleic acid species. Here we demonstrate that intracellular targeting of these molecules elicits an innate immune response and define viral-related RNA:DNA hybrid sequences that are sensed by both plasmacytoid and conventional dendritic cells (DCs). Finally, we identify MyD88 and TLR9 as the signalling adaptor and sensor required for this response, thereby establishing RNA:DNA hybrids as novel high-affinity ligands for TLR9.

## Results

### Synthesis and purification of RNA:DNA hybrids with viral sequence motifs

**Figure 1:**
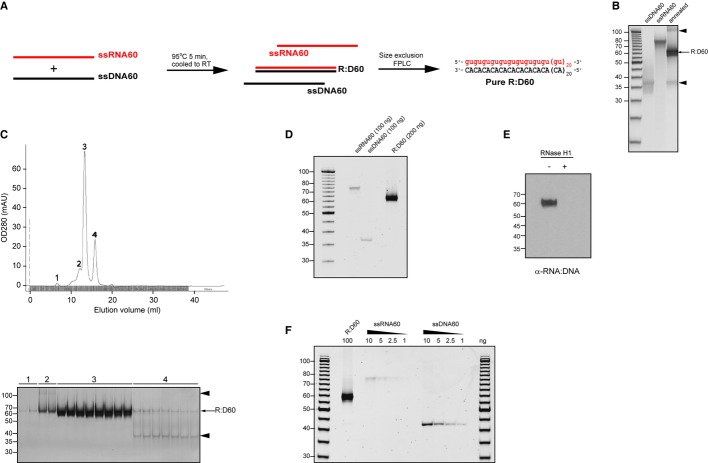
Generation of purified RNA:DNA hybrids.
Schematic representation of the synthesis and purification of the 60-bp RNA:DNA hybrid “R:D60”. Equimolar amounts of single-stranded RNA and DNA oligonucleotides were heat-denatured and gradually cooled to form RNA:DNA hybrids. Contaminating nucleic acids were removed by size-exclusion FPLC.Annealing of ssDNA60 and ssRNA60 oligonucleotides generates a 60-bp RNA:DNA hybrid with low levels of contaminating nucleic acid by-products. Native PAGE analysis of 100 ng of each single-stranded oligonucleotide prior to hybridisation and 200 ng post-annealing. Contaminating nucleic acids are indicated by arrowheads.FPLC gel filtration separates R:D60 from contaminating nucleic acids. Top, OD_280_ readings of eluted fractions. Bottom, analysis of 100 μl of selected FPLC fractions (peaks 1–4) by native PAGE. Contaminating nucleic acids are indicated by arrowheads.FPLC-purified R:D60 is free from contaminating nucleic acids. Native PAGE analysis of concentrated R:D60 from “peak 3” fractions alongside single-stranded constituent oligonucleotides.Purified R:D60 is an RNA:DNA hybrid as demonstrated by immunoblotting with an RNA:DNA hybrid-specific antibody. Immunoblotting with the S9.6 monoclonal antibody detects intact R:D60 but not R:D60 that has been enzymatically digested by the RNA:DNA hybrid-specific enzyme RNase H1.Native PAGE analysis is sensitive enough to detect ≤ 1% ssDNA and ≤ 2.5% (w/w) ssRNA within the purified R:D60 hybrid.

To address whether RNA:DNA hybrids represent a novel class of molecular pattern sensed by PRRs, we generated a synthetic 60-bp hybrid containing a repetitive guanosine-uridine-(GU) RNA strand motif (Fig 1A), on the basis that GU-rich viral RNA sequences are established nucleic acid ligands (Diebold *et al*, 2004; Heil *et al*, 2004). Chemically-synthesised oligonucleotides were annealed to form a 60-bp duplex (“R:D60”) and hybridrisation confirmed by native polyacrylamide gel electrophoresis (PAGE) analysis (Fig 1B). However, low levels of contaminating nucleic acid species were also evident, including one with identical electrophoretic mobility to the constituent DNA oligonucleotide (ssDNA60) and also a high-molecular-weight species, likely to represent a multimeric form of the hybrid (Fig 1B, arrowheads). Fast performance liquid chromatography (FPLC) was used to remove the potentially immunostimulatory by-products by size-exclusion fractionation (Fig 1C). This resulted in a pure nucleic acid species of 60 bp (Fig 1D), which was confirmed to be an RNA:DNA hybrid by immunoblotting with the RNA:DNA hybrid-specific S9.6 monoclonal antibody (Boguslawski *et al*, 1986) and enzymatically by exhibiting sensitivity to RNase H (Fig 1E). Additional nucleic acid species were undetectable by PAGE analysis (Fig 1F), establishing that the purity of R:D60 was ≥ 97.5%, given that 1% ssDNA and 2.5% (w/w) ssRNA could be visualised by this method. Using this strategy, sufficient R:D60 was then purified for subsequent experiments to investigate whether RNA:DNA hybrids stimulate an innate immune response.

### Dendritic cells are phenotypically activated and secrete cytokines in response to intracellular RNA:DNA hybrids

**Figure 2:**
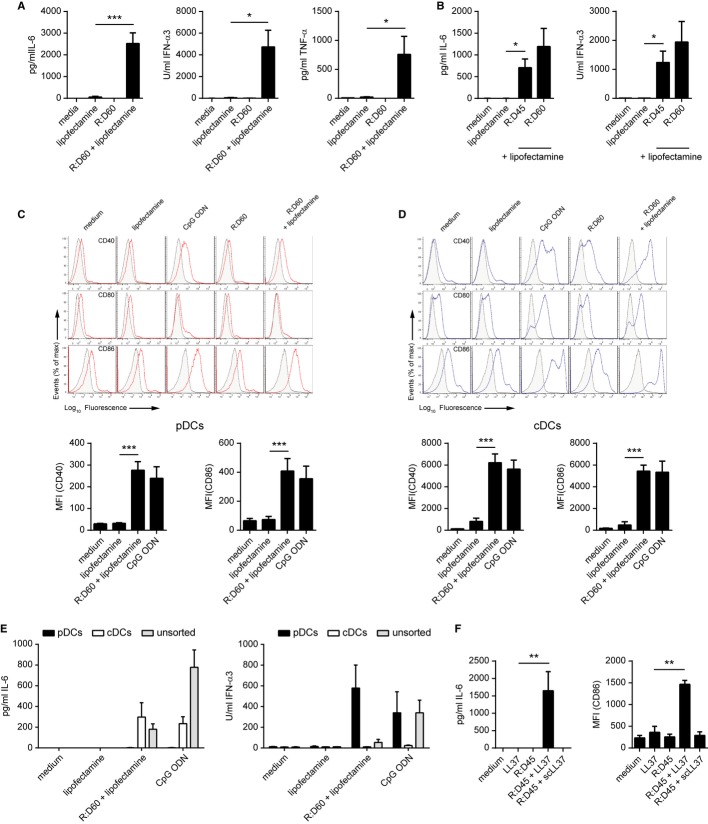
Intracellular RNA:DNA hybrids induce cytokine production and activation of dendritic cells. A Transfection of R:D60 into FLDCs stimulates IL-6, IFN-α and TNF-α secretion. Bone marrow-derived FLDC cultures were transfected with R:D60 complexed with Lipofectamine LTX. Supernatant cytokine levels were quantified 18 h later by ELISA. Data shown are the mean of at least five independent experiments ± s.e.m. ***P = 0.001023 (IL-6), *P = 0.015561 (IFN-α), *P = 0.45789 B Stimulation of cytokine production in FLDCs by a 45-bp RNA:DNA hybrid containing sequence from the HIV-1 gag gene. FLDCs were transfected with R:D45 or R:D60 using Lipofectamine LTX. Data shown are from four independent experiments ± s.e.m.; *P =* *0.0122 (IL-6), *P =* *0.0201 (IFN-α). C, D R:D60 induces phenotypic activation of FLDCs. Unsorted FLDC cultures were stimulated with R:D60 as described for (A). Expression levels of the co-stimulatory molecules CD40, CD80 and CD86 on CD11c^+^ B220^+^ pDCs (C) and CD11c^+^ B220^−^ cDCs (D) were quantified by flow cytometry. Below, median fluorescence intensity (MFI) values for CD40 and CD86 from six independent experiments ± s.e.m.; ***P =* *0.000003 (pDCs CD86), ***P =* *0.0001 (pDCs CD40), ***P =* *0.00009 (cDCs CD40), ***P =* *0.000000003 (cDCs CD86). CpG ODN added to the culture medium was included as a control. (CpG A could be added to cultures without complexing to Lipofectamine, as it generates large macromolecular aggregates due to unusual self-aggregating properties, sufficient to stimulate spontaneous cellular uptake (Wu *et al* 2004)). E R:D60 stimulates IL-6 secretion by cDCs and IFN-α production by pDCs. Day 8-FLDC cultures were sorted into CD11c^+^ B220^+^ PDCA1^+^ (pDC) and CD11c^+^ B220^−^ PDCA1^−^ (cDC) populations and stimulated as described for (C) and (D). Cytokine concentrations were quantified 18 h later by ELISA. Data shown are from three (IL-6) or four (IFN-α) independent experiments ± s.e.m. F R:D45 delivered into the cell by LL-37 stimulates cytokine production and phenotypic activation of FLDCs. 1 μg/ml R:D45 complexed with 25 μg/ml LL-37 or scrambled LL-37 (scLL-37) peptide was added to the medium of FLDC cultures. Supernatant cytokine levels and expression of co-stimulatory molecules were determined. Data shown are representative of three independent experiments ± s.e.m. (*n* = 3 replicates); **P =* *0.0025 (CD86), **P =* *0.0090 (IL-6).

Nucleic acid-sensing PRRs of the innate immune system are widely expressed across a range of cell types, including non-immune cells such as fibroblasts. Therefore R:D60 was initially transfected into primary mouse embryonic fibroblasts (MEFs). However, this failed to induce the expression of genes encoding type I IFNs, in contrast to the robust response induced by the TLR3 ligand poly(I:C) and the TLR9 ligand CpG ODN (ODN1585, Type A CpG ODN) (Supplementary Fig S1A). This led us to consider whether the detection of RNA:DNA hybrids might be restricted to specialised APCs. Transfection of R:D60 into bone-marrow derived macrophages (BMDMs) failed to induce the secretion of a range of cytokines by these cells, including IFN-α, IL-6 and TNF-α (Supplementary Fig S1B). In contrast, transfection of R:D60 into Fms-like tyrosine kinase 3-ligand (Flt-3)-differentiated bone marrow-derived dendritic cells (FLDCs) resulted in substantial production of IL-6 (*P* = 0.0010), IFN-α (*P* = 0.0156) and TNF-α (*P* = 0.0458) (Fig 2A). To confirm that viral RNA:DNA hybrids were immunostimulatory, we generated a second RNA:DNA hybrid containing 45 bp of the HIV-1 group-associated antigen (gag) gene (“R:D45”) (Supplementary Fig S2). Transfection of this hybrid into FLDCs also induced a substantial inflammatory cytokine response, comparable to that stimulated by R:D60 (Fig 2B).

FLDCs are a heterogeneous population that can be subdivided into conventional DC (cDC) and plasmacytoid DC (pDC) populations by surface expression of B220/CD45R (Brasel *et al*, 2000; Brawand *et al*, 2002), which differentially sense viral nucleic acids (Kato *et al*, 2005). We therefore investigated which DC subtype was responding to RNA:DNA hybrids. Transfection of R:D60 into FLDCs induced phenotypic activation of both FACS-purified pDC (CD11c^+^ B220^+^) and cDC (CD11c^+^ B220^−^) populations, as determined by upregulated surface expression of the costimulatory molecules CD40, CD80 and CD86 (Fig 2C, D). Next, we sought to determine which subset of FLDCs was responsible for the cytokine response to intracellular R:D60, as phenotypic activation can occur in the absence of cytokine production. FLDCs were sorted into pDC (CD11c^+^ B220^+^ PDCA-1^+^) and cDC (CD11c^+^ B220^−^ PDCA-1^−^) populations by flow cytometry and transfected with R:D60. This induced a pro-inflammatory cytokine response (IL-6) exclusively in cDCs (Fig 2E, left panel) whereas type I IFN (IFN-α) was produced solely by pDCs (Fig 2E, right panel).

The complexing of R:D60 with a liposomal transfection reagent (Lipofectamine) was essential for cytokine production, in keeping with intracellular detection or RNA:DNA hybrids by PRRs (Fig 2A). LL-37, a naturally occurring inflammatory product of neutrophils, epithelial cells and macrophages (Beaumont, 2013), is known to bind and internalise nucleic acids into mammalian cells (Sandgren *et al*, 2004; Lande *et al*, 2007; Ganguly *et al*, 2009; Lai *et al*, 2011). Internalisation of R:D45 using LL-37 but not a scrambled peptide control (scLL37) was also able to induce cytokine production and activation of FLDCs (Fig 2F).

We therefore concluded that intracellular RNA:DNA hybrids induce cytokine secretion and phenotypic activation of dendritic cells *in vitro*.

### Intracellular targeting of RNA:DNA hybrids stimulates cytokine secretion *in vivo* in mice and *ex vivo* in human PBMCs

**Figure 3:**
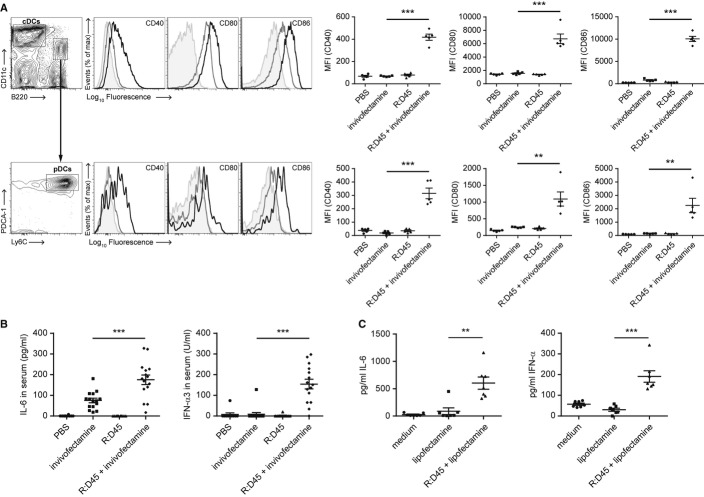
R:D45 activates DCs and induce a systemic cytokine response *in vivo* in mice and *ex vivo* in human cells.
Delivery of R:D45 complexed to Invivofectamine *in vivo* phenotypically activates DCs. C57BL/6 mice were injected intraperitoneally with 80 μg R:D45 or 80 μg R:D45 complexed to Invivofectamine and the activation of splenic DC populations was analysed 12 h later by flow cytometry. Left, representative histograms comparing cell surface expression of the indicated marker on DCs from mice treated with Invivofectamine alone (grey) and R:D45 complexed to Invivofectamine (black). Isotype control shaded grey. Right, MFI values for CD40 (*P* =* *0.0000013), CD80 (*P* =* *0.000106) and CD86 (*P* =* *0.00000024). Data shown are from one experiment ± s.e.m. (*n* = 5 mice per group), representative of a total of three independent experiments. Large-scale R:D45 hybrid synthesis was performed for each experiment, with purity estimated by PAGE at 96%, 91%, and 98% hybrid, respectively.R:D45 complexed to Invivofectamine induces cytokine production *in vivo*. C57BL/6 mice were injected with R:D45 as described for (A). Serum levels of IL-6 (***P =* *0.000526) and IFN-α (***P =* *0.0000026) were determined 12 h post-injection. Data pooled from three independent experiments ± s.e.m. (10–15 mice total per condition).R:D45 induces cytokine production when transfected *ex vivo* in human PBMCs. Freshly isolated PBMCs were transfected with R:D45 complexed to Lipofectamine LTX. Supernatant cytokine levels were quantified 18 h later by ELISA. Data pooled from two independent experiments ± s.e.m., **P =* *0.00166 (IL-6), ***P =* *0.00011 (IFN-α) (7 donors in total).

As FLDC cultures represent an *in vitro* model of steady-state splenic DC populations (Brawand *et al*, 2002; Naik *et al*, 2005) we next investigated whether RNA:DNA hybrids could be detected by splenic DCs *in vivo*. R:D45 was injected intraperitoneally into C57BL/6 mice either alone or complexed to the cationic liposome Invivofectamine. Analysis of splenic DC populations 12 h post injection by flow cytometry showed a significant upregulation of CD40, CD80 and CD86 expression by cDCs and pDCs when the hybrid was administered in a liposomal complex (Fig 3A). A comparable level of activation was seen between cDC subsets (Supplementary Fig S3). Furthermore, R:D45 complexed to Invivofectamine induced a robust cytokine response, with significantly elevated levels of both IL-6 and IFN-α in the serum of these mice (Fig 3B). Consistent with *in vitro* FLDC experiments, liposomal delivery was essential for RNA:DNA hybrid stimulation for cytokine secretion and DC activation (Fig 3A, B).

To investigate whether RNA:DNA hybrids were also able to induce a cytokine response in human cells, we used *ex vivo* peripheral blood mononuclear cells (PBMCs) that comprise a mixed population of cells including lymphocytes, monocytes, cDCs and pDCs. Transfection with R:D45 induced significant production of both IL-6 and IFN-α by PBMCs (Fig 3C), establishing that the innate immune sensing of RNA:DNA hybrids is not species-specific.

In summary we concluded that the detection of RNA:DNA hybrids within an intracellular compartment occurs in mice (*in vivo*) and in humans (*ex vivo*). Consequently, we next sought to identify the cellular pathway involved in the sensing of RNA:DNA hybrids.

### MyD88 is essential for FLDC activation by RNA:DNA hybrids

**Figure 4:**
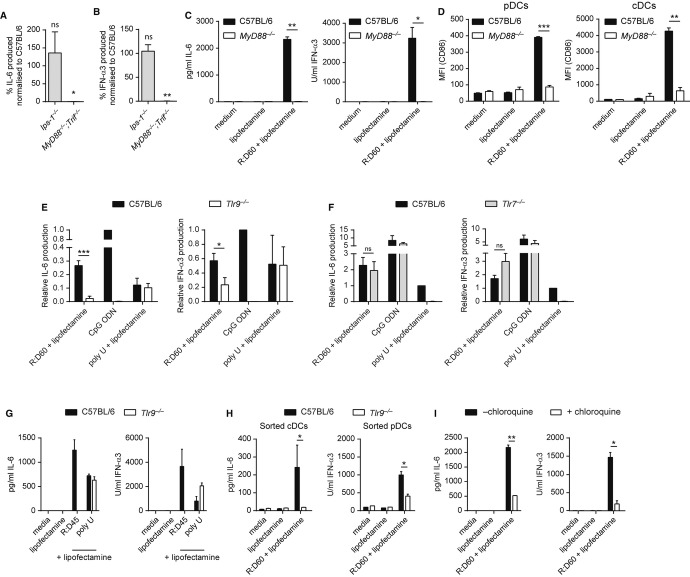
Detection of RNA:DNA hybrids requires MyD88 and TLR9. A, B The cytokine response to R:D60 is absent in *Myd88*^−/−^*Trif*^−/−^ but not *Ips-1*^−/−^ mice. FLDCs derived from *MyD88*^−/−^*;Trif*^−/−^, *Ips-1*^−/−^ and wild-type (C57BL/6) control mice were transfected with R:D60. Supernatant levels of IL-6 (A) and IFN-α (B) are represented as percentage of cytokine produced by C57BL/6 wild-type controls included in each experiment. Data shown are the mean of three (*Ips-1*^−/−^) or two (*Myd88*^−/−^*;Trif*^−/−^) independent experiments ± s.e.m. (one-sample *t*-test). ns *P* = 0.9442 (IPS-1 IFN-α), **P = 0.0102 (MyD88/TRIF IFN-α), ns P = 0.5790 (IPS-1 IL6), *P = 0.0396 (MyD88/TRIF IL6). C, D MyD88 is essential for cytokine secretion and phenotypic activation of FLDCs by R:D60. FLDCs derived from *MyD88*^−/−^ and C57BL/6 control mice were transfected with R:D60. Supernatant cytokine levels (C) and surface expression of co-stimulatory molecules were determined 18 h post-transfection, with CD86 shown as representative (D). Data shown are the mean of 2 independent experiments ± s.e.m. (C) or ± s.d. (D) (unpaired *t*-test). In (C), **P =* *0.0015 (IL-6); *P =* *0.0271 (IFN-α). In (D): ***P =* *0.00072 (pDCs CD86); **P =* *0.00444 (cDCs CD86). E, F The cytokine response to R:D60 is significantly impaired in TLR9-deficient but not TLR7-deficient FLDCs. Cultures derived from *Tlr9*^−/−^, *Tlr7*^−/−^ and C57BL/6 mice were transfected with R:D60/poly U, or stimulated by the addition of CpG ODN to the culture medium. Supernatant cytokine levels were determined by ELISA. Levels of IL-6 and IFN-α3 are represented as percentage of cytokine produced by wild-type controls to the TLR9 ligand CpG ODN (E) or the TLR7 ligand poly U (F). Data are the mean of six and three independent experiments ± s.e.m., respectively. ***P = 0.0003 (*Tlr9*^−/−^ IL-6), *P = 0.0514 (*Tlr9*^−/−^ IFN-α), ns *P* = 0.3761 (*Tlr7*^−/−^ IL-6), ns *P* = 0.1224 (*Tlr7*^−/−^ IFN-α). G R:D45-induced cytokine production is TLR9-dependent. FLDCs derived from *Tlr9*^−/−^ and C57BL/6 mice were transfected with R:D45 or poly U. Cytokine levels were determined 18 h post-transfection. Phenotypic activation as determined by CD40/80/86 expression was also entirely TLR9-dependent. Data are representative of two independent experiments ± s.d. of duplicate samples. H Cytokine production by both cDCs and pDCs in response to R:D60 is impaired in *Tlr9*-deficient FLDCs. Day 8-FLDC cultures were sorted into CD11c^+^ B220^+^ PDCA1^+^ (pDC) and CD11c^+^ B220^−^ PDCA1^−^ (cDC) populations and transfected with R:D60 or stimulated by the addition of CpG ODN. Data shown are representative of three independent experiments ± s.d. of replicate samples. *P =* *0.0355 (cDCs, IL-6), *P =* *0.0165 (pDCs, IFN-α). I The cytokine response to R:D60 is sensitive to chloroquine treatment. FLDCs derived from C57BL/6 mice were treated with 10 μM chloroquine prior to stimulation with R:D60 and CpG ODN as described for (H). Data shown are representative of three independent experiments ± s.d. of replicate samples. **P =* *0.0025 (IL-6), *P =* *0.0151 (IFN-α).

Many nucleic acid-sensing PRRs require the binding of an adaptor molecule to mediate downstream signalling and subsequent cytokine production. To identify the PRR-adaptor pathways sensing RNA:DNA hybrids, FLDCs derived from mice lacking the adaptor proteins IPS-1, TRIF or MyD88 were transfected with R:D60. The cytokine response of *Ips-1*^−/−^ FLDCs was indistinguishable from that of C57BL/6 control FLDCs, however R:D60-induced production of both IL-6 and IFN-α was undetectable in cells from mice lacking both the TRIF and MyD88 adaptor molecules (Fig 4A, B). FLDCs deficient in MyD88 alone failed to produce cytokines in response to R:D60 (Fig 4C), while cytokine production was intact in *Trif*^−/−^ FLDCs (Supplementary Fig S4A). Similarly, phenotypic activation of both pDCs and cDCs was abrogated in cells lacking MyD88 (Fig 4D) but intact in *Trif*^−/−^ FLDCs (Supplementary Fig S4B), thereby confirming that MyD88 is essential for downstream signalling following PRR detection of RNA:DNA sensing in both DC subtypes.

### TLR9 senses RNA:DNA hybrids

Given that response to RNA:DNA hybrids was independent of TRIF, TLR3 and the DDX1/DDX21/DHX36 complex (Yamamoto *et al*, 2002; Zhang *et al*, 2011) were ruled out as candidate sensors of these hybrids. Likewise, the cytosolic RNA sensors RIG-I and MDA5 were excluded as both are dependent on binding to IPS-1 for downstream signalling (Kawai *et al*, 2005). However, TLR7 and TLR9 both require MyD88 for downstream signalling (Schnare *et al*, 2000; Hemmi *et al*, 2002) and so represented strong candidates for the intracellular sensor of RNA:DNA hybrids.

FLDCs derived from *Tlr7*^−/−^ and *Tlr9*^−/−^ mice were transfected with R:D60. Production of both IL-6 and IFN-α was found to be significantly reduced in *Tlr9*^−/−^ FLDCs (Fig 4E). Similarly, cytokine production in response to R:D45 was undetectable (Fig 4G). Conversely, *Tlr7*^−/−^ FLDCs displayed normal cytokine responses to RNA:DNA hybrids (Fig 4F). Analysis of cytokine production in FACS-sorted FLDCs confirmed that IL-6 production by TLR9-deficient cDCs was completely abolished and IFN-α production by pDCs significantly impaired (Fig 4H). Therefore, TLR9 appears to be the sole RNA:DNA hybrid-sensing receptor in cDCs and represents the major receptor for hybrids in pDCs. Residual IFN-α secretion by *Tlr9*^−/−^ pDCs in response to R:D60 could suggest an additional hybrid-sensing receptor in this cell type, in which case DHX9 or DHX36 could be plausible candidates given that they have been reported to be MyD88-dependent sensors (Kim *et al*, 2010).

Chloroquine treatment of wild-type C57BL/6 FLDCs also impaired cytokine secretion following subsequent R:D60 transfection (Fig 4I). Since cholorquine is an established inhibitor of endosomal TLR-nucleic acid binding (Hacker *et al*, 1998) this indicated that hybrids activate TLR9 in an endosomal compartment.

Together, these data established that endosomal detection of RNA:DNA hybrids by TLR9 leads to activation of downstream signalling and cytokine production.

### Intact RNA:DNA hybrids stimulate cytokine secretion by FLDCs

**Figure 5:**
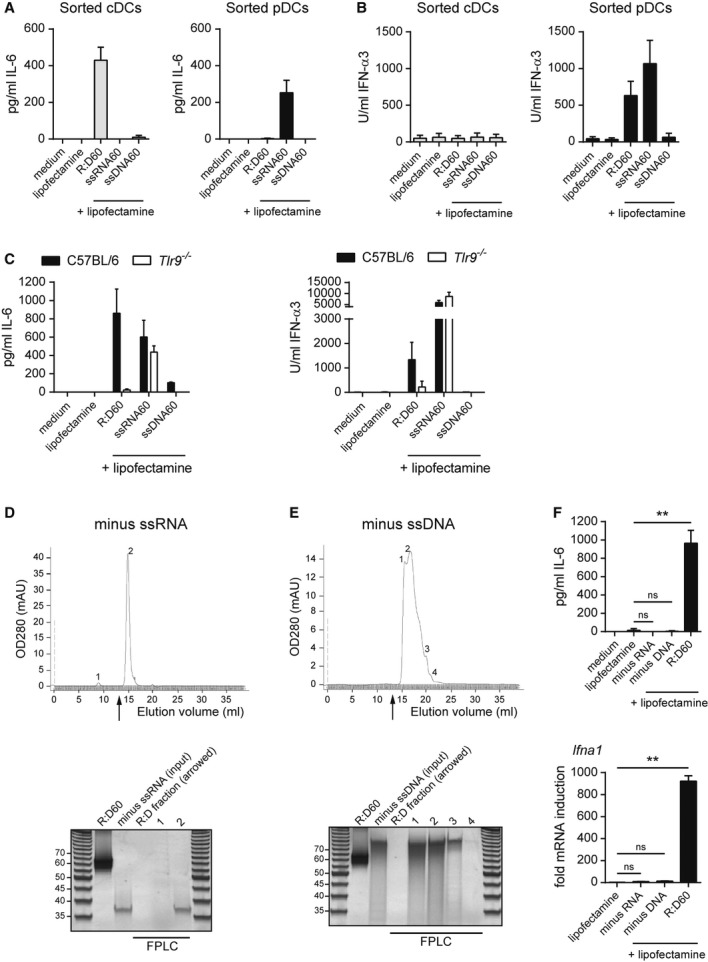
TLR9 is activated by intact RNA-DNA hybrids. A, B The cytokine response (IL-6 (A), IFN-α (B)) of FLDCs to R:D60 is distinct from that of its single-stranded nucleic acid components. Day 8-FLDC cultures were FACS-sorted into pDC and cDC populations and transfected with R:D60 or equimolar amounts of ssRNA60 and ssDNA60. Data shown are the mean of three independent experiments ± s.e.m. C Cytokine induction by R:D60 but not ssRNA60 is TLR9-dependent. FLDCs derived from *Tlr9*^−/−^ and C57BL/6 mice were transfected as described for (A) and (B). Data shown are from two independent experiments ± s.d. D, E The cytokine response of FLDCs to R:D60 is not due to immunostimulatory contaminants remaining after FPLC. Nucleic acid hybridisation reactions were generated as outlined in Fig 1A omitting one of the two oligonucleotides and fractionated using identical FPLC conditions to those used to purify annealed RNA:DNA hybrids. Upper panel: nucleic acids from mock hybridization reactions (minus ssRNA (D), minus ssDNA (E)) eluted later than the established elution position of R:D60 (arrow). The fractions (12.9 ml – 13.7 ml) corresponding to those that would usually contain the hybrid (“R:D fraction”) and fractions containing the nucleic acids at the indicated peaks, were concentrated by ethanol precipitation. Lower panels: 200 ng of FPLC-purified R:D60, 200 ng of hybridisation reaction column input (labelled “minus ssRNA” and “minus ssDNA” respectively), along with equal volumes of each fraction, were analysed by native PAGE. F Immunostimulatory contaminants are absent from the R:D60 fraction. FLDCs transfected with FPLC-purified R:D60 and the equivalent volume of R:D fractions from the hybridisations shown in (D) and (E) using Lipofectamine 2000. Supernatant concentrations of IL-6 (upper panel) were quantified 18 h post-transfection by ELISA and *Ifna1* transcript levels (lower panel) normalised to *Actb* expression quantified 6 h post-transfection by qRT-PCR (fold mRNA induction from medium alone samples). Data shown are the mean of three experiments ± s.e.m. (IL-6, **P =* *0.0028) and PCR triplicates ± s.d. (*Ifna1*, **P =* *0.0001).

Single-stranded DNA (ssDNA) can potently activate TLR9-mediated signalling (Hemmi *et al*, 2000), therefore the degradation of RNA:DNA hybrids by intracellular RNase H enzymes to ssDNA could provide an explanation for TLR9 activation by intracellular RNA:DNA hybrids. To address this, we transfected equimolar amounts of the constituent 60-mer single-stranded oligonucleotides into FACS-sorted FLDC populations and measured cytokine production. Transfection of the DNA strand (“ssDNA60”) resulted in the production of only low levels of IL-6 by cDCs (Fig 5A) and no IFN-α secretion by pDCs (Fig 5B). Therefore, ssDNA generated from the enzymatic digestion of R:D60 is unlikely to be responsible for the activation of TLR9-dependent signalling.

We also considered the possibility that internalised RNA:DNA hybrids could be unwound by cytosolic helicases, generating ssRNA in addition to ssDNA. Although transfection of the RNA strand (“ssRNA60”) induced a robust cytokine response in FACS-sorted cDCs and pDCs, the nature of this response differed from that induced by R:D60; as it was restricted to pDCs (Fig 5A, B) and independent of TLR9 (Fig 5C).

We therefore concluded that TLR9 senses intact RNA:DNA hybrids without enzymatic processing or physical dissociation of the heteroduplex, strongly suggesting that RNA:DNA hybrids directly bind TLR9.

We then sought to confirm that cytokine production by FLDCs was due to the RNA:DNA hybrids themselves, rather than it being attributable to a contaminant introduced during synthesis or transfection. Firstly, contamination of the Lipofectamine transfection reagent was excluded as it did not induce cytokine production or phenotypic activation of FLDCs (Fig 2). Secondly, in the absence of transfection reagent, R:D60 was not immunogenic, thereby excluding endotoxin contamination in the hybrid preparation (Fig 2). Thirdly, to exclude the possibility that low levels of single-stranded nucleic acids present in the hybrid preparation below the sensitivity of PAGE analysis could represent a source of contamination, the FPLC process was repeated using “mock” hybridisation reactions in which one of the constituent oligonucleotides (ssRNA60 or ssDNA60) was omitted. Following FLPC, nucleic acids were not visualised in the elution fractions from mock hybridisations, that usually would have contained the hybrid (Fig 5D, E). Furthermore, these fractions were not immunostimulatory when transfected into FLDCs (Fig 5F). Therefore, FLDC activation could not be attributed to the co-filtration of non-visible contaminants in FPLC-purified R:D60.

**Figure 6:**
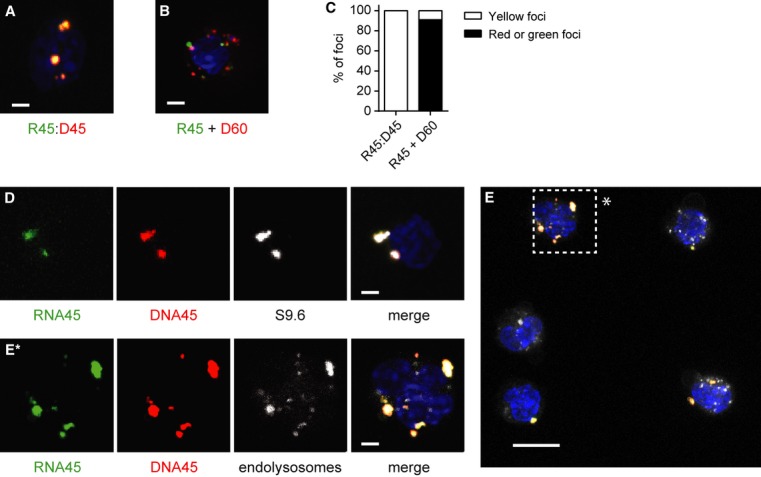
Intact RNA:DNA hybrids are detectable by immunofluorescence inside FLDCs.
Fluorescently labelled RNA and DNA strands of R:D45 remain equally colocalised in FLDCs following transfection, but non-complementary RNA and DNA oligonucleotides do not.Confocal maximum projection image of a cell after transfection with fluorescently labelled R:D45, generated by annealing Cy5-labelled ssRNA45 and Cy3-labelled ssDNA45 oligonucleotides. DNA visualised by DAPI, blue.Confocal image of a cell transfected with non-complementary Cy3-ssRNA45 and Cy5-ssDNA60 oligonucleotides.Quantification of nucleic acid foci in FLDCs transfected with either fluorescently labelled R:D45 (*n* = 50) or Cy3-ssRNA45 with Cy5-ssDNA60 (*n* = 118).Intracellular R:D45 is detected by the anti-RNA:DNA hybrid antibody S9.6. FLDCs were transfected with fluorescently labelled R:D45 as for (A), and fixed and permeabilised prior to immunofluorescent detection with the S9.6 antibody. Representative maximum projection confocal image is shown. Scale bar, 2 μm.R:D45 frequently colocalises with the endolysosomal marker LysoTracker. Cells were transfected with fluorescently labelled R:D45 as for (A) then incubated with LysoTracker for 1 h. Maximum projection image of a representative field of view. Scale bar, 10 μm. E^*^ Higher magnification image of a cell (*) in (E). Scale bar, 2 μm.

To directly establish that RNA:DNA hybrids remained intact within FLDCs, we fluorescently labelled 45-bp RNA:DNA hybrids (R:D45) by conjugation of Cy5 and Cy3 fluorophores to the 5′ ends of the ssRNA45 and ssDNA45 oligonucleotides, respectively. Visualisation of the intracellular nucleic acids post-transfection using confocal microscopy established that Cy3 and Cy5 signals remained colocalised in all foci with equivalent intensity, consistent with the R:D45 hybrid remaining intact within the cell (Fig 6A, yellow foci). As a control, the non-complementary oligonucleotides Cy3-ssRNA45 and Cy5-ssDNA60 were “annealed” in the same manner and transfected into FLDCs. In this case Cy3 and Cy5 signals did not show the same level of colocalisation (Fig 6B), with only 9% of foci showing similar intensity Cy3 and Cy5 signals, suggesting a random distribution of non-hybridised oligonucleotides in intracellular foci (Fig 6B, C). Furthermore, immunostaining with the S9.6 antibody also detected fluorescently labelled R:D45 foci, confirming that these represent intact intracellular RNA:DNA hybrids (Fig 6D).

As Lipofectamine-mediated uptake of nucleic acid-liposomal complexes initially occurs via endocytosis (Akita *et al*, 2004) we also examined whether fluorescently labelled R:D45 could colocalise with LysoTracker, a marker of acidified endolysosomes. Confocal microscopy demonstrated that many Cy3/Cy5-labelled R:D45 foci colocalised with LysoTracker (Fig 6E, Supplementary Fig S5), establishing that liposomal mediated transfection delivers RNA:DNA hybrids to the appropriate intracellular compartment where TLR9 sensing occurs.

### TLR9 directly binds RNA:DNA hybrids with a high affinity

**Figure 7:**
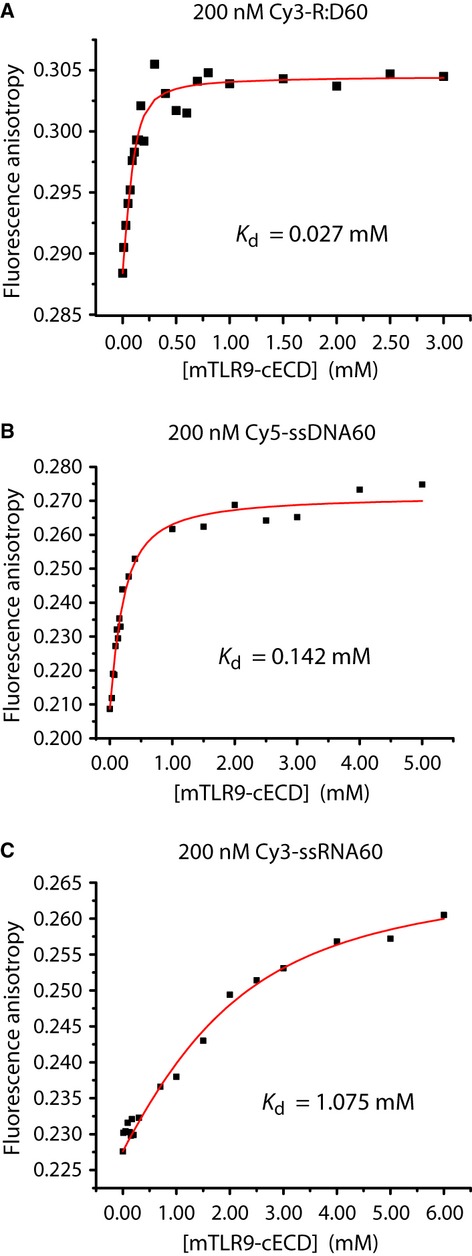
TLR9 directly binds RNA:DNA hybrids. A–C Equilibrium binding of the C-terminal proteolytic cleavage fragment of mouse TLR9 ectodomain (mTLR9-cECD) to R:D60 measured by fluorescence spectroscopy. The 3′ end of the RNA strand of R:D60 was labelled with a Cy3 fluorophore for this experiment, and was at a concentration of 200 nM (A). The binding curve was fitted as described in the Materials and Methods giving a *K*_d_ value of 27.2 nM for mTLR9-cECD binding to Cy3-R:D60. A control for non-specific binding using bovine serum albumin instead of mTLR9-cECD showed no binding to Cy3-labelled R:D60. Equilibrium binding of 5′ Cy5-labelled ssDNA60 (*K_d_* = 142 nM) (B) and 5′ Cy3-labelled ssRNA60 to mTLR9-cECD (*K_d_* = 1075 nM) (C) were also quantified.

To determine whether TLR9 and R:D60 interact in the absence of other factors and to quantify the affinity of the interaction, binding of purified recombinant TLR9 to fluorescently labelled RNA:DNA hybrid was assessed. Since the ectodomain of TLR9 is proteolytically cleaved by endosomal proteases to produce an active receptor (Ewald *et al*, 2008; Park *et al*, 2008), the affinity of the C-terminal proteolytic cleavage fragment of mouse TLR9 ectodomain (mTLR9-cECD) for R:D60 was measured by fluorescence anisotropy polarization. The dissociation equilibrium constant (*K*_d_) was determined by titrating mTLR9-cECD into a solution of R:D60 labelled at the 5′ end of the RNA strand with the cyanine Cy3 dye. R:D60 binding to mTLR9-cECD was unexpectedly strong, with a *K*_d_ value of 0.027 μM (Fig 7A), revealing an interaction approximately 25-fold stronger than between mTLR9-cECD and prototypical single-stranded DNA TLR9 ligands (Li *et al*, 2012). This was also stronger than a previously tested dsDNA ligand (Li *et al*, 2012). Significantly, the affinity for the single-stranded constituents of R:D60 was also much lower with *K*_d_ of 0.136 and 1.075 μM for ssDNA60 and ssRNA60 respectively (Fig 7B, C).

### RNA:DNA hybrids accumulate in the cytosol and endosomes during retroviral infection

**Figure 8:**
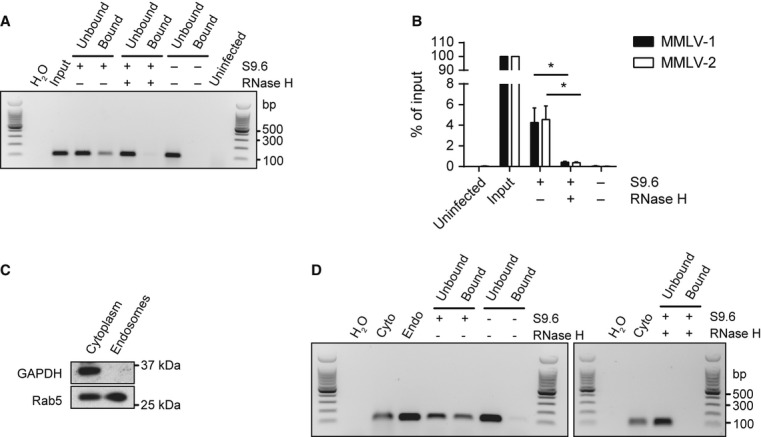
Viral RNA:DNA hybrids accumulate in the cytoplasm and endosomes of MMLV infected cells. A, B Viral RNA:DNA hybrids are present in the cytoplasm of MMLV infected cells. (A) MMLV-specific PCR detects RNA:DNA hybrids immunoprecipitated from cytoplasmic nucleic acids. As PCR detects both MMLV DNA and RNA:DNA hybrids, specificity of the S9.6 pull down for hybrids is confirmed by the absence of PCR product in control samples processed with either S9.6 antibody omitted or when cytoplasmic nucleic acids were pretreated with RNase H. Labels: H_2_O, PCR without template added; Input, cytoplasmic nucleic acids prior to immunopreciptation (equal in proportion to post-pull down template); Uninfected, cytoplasmic nucleic acids from uninfected B3T3 cells. B Quantification of MMLV RNA:DNA hybrids immunoprecipitated by S9.6 by qPCR using two independent MMLV primer sets. S9.6 immunoprecipitate contains 4.1 ± 1.1% of input molecules. RNase H treatment reduces the amount of MMLV-DNA pulled down by S9.6 by >90%. Data shown are the mean of three independent IP/qPCR experiments ± s.d. *P = 0.0366 (MMLV-1), *P = 0.231 (MMLV-2). C Validation of endosomal fractionation: the early endosome marker Rab5 is enriched in endosomal preparations. Western blotting of cytoplasmic and endosomal fractions shows the presence of the endosomal marker Rab5 in both endosomal and cytoplasmic fractions, whereas GAPDH is only present in cytoplasmic fractions. Densitometry measurements show that relative Rab5 enrichment is > 22-fold relative to GAPDH. D Viral RNA:DNA hybrids are present in endosomal fractions of MMLV-infected cells. MMLV DNA was detected by PCR after S9.6 pull-down of hybrids from endosomal nucleic acids, but not in beads only or RNase H treated controls.

Many pathogens, most notably retroviruses, generate RNA:DNA hybrids as replication intermediates within an infected cell. To establish if significant levels of intact RNA:DNA hybrids were present within infected cells, we used the S9.6 antibody to affinity-purify RNA:DNA hybrids from B3T3 fibroblasts infected with the retrovirus Moloney Murine Leukaemia Virus (MMLV). Following S9.6 pull down of RNA:DNA hybrids from cytoplasmic extracts of infected cells, viral nucleic acid was detectable by PCR using virus-specific primers (Fig 8A, B). As PCR detects both MMLV DNA and RNA:DNA hybrids, the specificity of the S9.6 pulldown for RNA:DNA hybrids was confirmed by pre-treatment with RNase H, which abrogated the PCR signal, consistent with pull down of intact RNA:DNA hybrids by the S9.6 antibody. Quantification by qPCR using two different sets of primers showed that S9.6 immunoprecipitates 4.1 ± 1.1% of MMLV cytoplasmic DNA (Fig 8B, *P* ≤ 0.03), demonstrating that significant levels of intact RNA:DNA hybrids can accumulate during viral infection. As detection of TLR9 ligands occurs in endosomes, we next sought to determine if viral RNA:DNA hybrids could be detected in the endosomes of infected cells. Endosomal fractions were prepared from B3T3 cells infected with MMLV by discontinuous sucrose gradient ultra-centrifugation and validated by immunoblotting to confirm the presence of the endosome marker Rab5, and absence of GAPDH, a cytosolic enzyme, from these fractions (Fig 8C). Subsequently, the S9.6 antibody was used to pull down RNase H-sensitive nucleic acids from this endosomal fraction (Fig 8D), consistent with the presence of viral RNA:DNA hybrids in the endosomes of MMLV-infected cells.

In summary, RNA:DNA hybrids bind directly to TLR9 with high affinity to activate the receptor and induce innate immune activation in DCs, indicating that intracellular RNA:DNA hybrids containing viral-related sequences are a novel class of immunostimulatory nucleic acid ligand. Taken together with the detection of RNA:DNA hybrids in endosomal fractions during a viral infection, these experiments also support the notion that endosomal sensing of RNA:DNA hybrids by TLR9 is physiologically relevant.

## Discussion

The definition of nucleic acid ligands for the PRRs of the innate immune system, such as di-cyclic GMP and 5′-triphosphate RNA, has led to important insights into the intracellular sensing of microbial pathogens over recent years (Myong *et al*, 2009; Schlee *et al*, 2009; Burdette *et al*, 2011). Here we report a novel nucleic acid ligand, establishing for the first time that RNA:DNA hybrids are a molecular pattern that induce a pro-inflammatory cytokine response in dendritic cells. Unexpectedly, RNA:DNA hybrids are sensed by TLR9, a PRR first characterised over a decade ago (Hemmi *et al*, 2000). Therefore, in addition to its established role in sensing unmethylated CpG-rich bacterial DNA, TLR9 also detects RNA-containing nucleic acids, thereby extending the role this receptor could play in viral sensing and systemic autoimmunity.

Previous studies of synthetic ligands for the cytosolic RNA helicase RIG-I have highlighted the importance of accurately defining the biochemical nature of nucleic acid ligands (Myong *et al*, 2009; Schlee *et al*, 2009). In our study, several lines of evidence permit us to conclude that intact RNA:DNA hybrids are TLR9 ligands. Firstly, our ligand is precisely defined, generated from chemically synthesised HPLC-purified oligonucleotides, and its purity demonstrated by PAGE and FPLC analysis. Secondly, robust activation of TLR9 requires the intact RNA:DNA hybrid, and this cannot be replicated by the constituent single-stranded nucleic acids. Finally, RNA:DNA hybrids directly bind the activated TLR9 receptor ectodomain with high affinity (Fig 7), with a much lower *K*_d_ than either its constituent single-stranded nucleic acid components, or known ssDNA TLR9 ligands measured under the same assay conditions (Li *et al*, 2012).

RNA:DNA hybrids represent an additional type of nucleic acid sensed by TLR9, distinct from the canonical unmethylated CpG-rich single-stranded DNA ligand (Hemmi *et al*, 2000; Bauer *et al*, 2001). However double-stranded DNA (dsDNA), the physiologically abundant form of DNA, is also a TLR9 ligand (Cornelie *et al*, 2004; Kindrachuk *et al*, 2007), therefore as duplex nucleic acids, hybrids are also plausible ligands for this receptor. Likewise, although CpG sequence motifs are classically associated with TLR9 binding (Hemmi *et al*, 2000; Bauer *et al*, 2001), such specificity is dependent on phosphorothiorate chemistry (Haas *et al*, 2008). This challenges the widely held and attractive assumption that unmethylated CpG-rich DNA is the natural TLR9 ligand, and so TLR9 activation by RNA:DNA hybrids without CpG sequences is also in keeping with existing literature. Furthermore, the phosphodiester backbone of the RNA:DNA hybrids used in this study is of a greater physiological relevance than CpG phosphorothiorate ODNs whose correspondence to natural ligands is limited both by strandedness and backbone structure.

The R:D60 RNA:DNA hybrid is notably more immunostimulatory than the corresponding ssDNA oligonucleotide at the same concentration (Fig 5A–C). A weak cytokine response at this relatively low concentration (25 nM) is in keeping with previous studies of phosphodiester ssDNA oligonucleotides (Yasuda *et al*, 2006; Haas *et al*, 2008), which demonstrated immunostimulation at higher concentrations. The differential response observed here correlates with the higher affinity of the TLR9 receptor for the hybrid (Fig 7), therefore binding affinity may be important in determining cytokine response. Other factors such as the relative stability of ssDNA and RNA:DNA hybrids within the endosome could also play a role. Future studies correlating the relative abundance and receptor affinities of naturally occurring TLR9 ligands with cytokine response may therefore be informative in defining which are most relevant to disease.

As RNA:DNA hybrids are essential intermediates in the replication of most microbial pathogens, perhaps most notably in the retroviral life cycle (Hu & Hughes, 2012), their detection by the innate immune system would be anticipated. Hybrid replication intermediates are also generated by DNA viruses (Pritchard *et al* 1998, Miller *et al* 1984) and so sensing of RNA:DNA hybrids could have broad utility for viral defence. Although TLR7 is the major sensor for retroviruses via detecting the ssRNA genome during viral entry (Kane *et al*, 2011; Lepelley *et al*, 2011), lentiviral replication intermediates can also be proinflammatory (Doitsh *et al*, 2010). Reverse transcription products containing RNA:DNA hybrids could be sensed by TLR9, which has been implicated in pDC sensing of lentiviruses (Mandl *et al*, 2008) and in suppressing endogenous retrovirus (ERV)-induced diseases via the modulation of humoral antibody responses (Yu *et al*, 2012).

It is conceivable that RNA:DNA hybrids could be delivered to endosomal TLR9 upon viral entry, as reverse-transcribed nucleic acids have been detected in retrovirions (Dornadula *et al*, 1999). Alternatively, as viral replication products, they may be transported through the endosomal pathway following autophagy (Lee *et al*, 2007). Most likely, death of virally infected cells with subsequent phagocytosis by APCs could result in presentation of RNA:DNA hybrids to TLR9. In this respect, it is of note that the complexing of RNA:DNA hybrids to LL37 efficiently activated DCs (Fig 2F), establishing at least one physiological mechanism by which hybrids released by dying cells can be delivered intracellularly to DCs. LL-37 has been shown to enhance TLR3 signalling in response to viral dsRNAs that are poor agonists by themselves (Lai *et al*, 2011) and it can also complex with self-DNA and self-RNA in autoimmune disorders, altering internalisation and endosomal processing to induce inflammatory responses to otherwise non-immunogenic nucleic acids via TLR7, TLR8 and TLR9 (Lande *et al*, 2007; Ganguly *et al*, 2009).

Systemically released RNA:DNA hybrids may likewise contribute to autoimmunity, analogous to the established importance of circulating DNA in SLE pathogenesis (Napirei *et al*, 2000; Yasutomo *et al*, 2001). While exogenous DNA is cleared by DNase I (Napirei *et al*, 2000), human serum lacks nuclease activity against RNA:DNA hybrids (R.E.R., unpublished data), so hybrids could persist extracellularly, thereby contributing to the activation of IFN-producing pDCs in lupus (Ronnblom *et al*, 2003). Alternatively, cell-intrinsic responses to self-RNA:DNA hybrids may result from the activation of endogenous retroviruses. Cytoplasmic nucleic acids encoded by retroelements accumulate in the *Trex1*^−/−^ mouse (Stetson *et al*, 2008) and result in systemic autoimmune disease (Gall *et al*, 2012). As mutations in the genes encoding both TREX1 and the trimeric RNase H2 complex cause autoinflammation in AGS (Crow *et al*, 2006a,b2006b), it has been proposed that RNase H2-AGS results from the failure to degrade the RNA:DNA intermediates of endogenous retroviruses (Stetson, 2009). Therefore, inappropriate activation of the innate immune response by endogenous RNA:DNA hybrids could be an important contributing factor in the pathogenesis of autoimmune disease (Supplementary Fig S7).

In conclusion, we demonstrate that RNA:DNA hybrids are novel molecular pattern that induce a TLR9-dependant innate immune response. This class of nucleic acids therefore warrants further investigation to establish its role in both autoimmunity and host response to viral infection. The identity of naturally occurring TLR9 microbial ligands has not yet been definitively established. Given that dsDNA and RNA:DNA hybrids coexist alongside other nucleic acids during pathogen infection, the precise nature of the substrates sensed by TLR9 remains an open question and an interesting future challenge.

## Materials and Methods

### Reagents

CpG-ODN (Mouse ODN1585, Human ODN2216, both type A CpG ODNs) were from Invivogen and poly U from Sigma Aldrich. Oligonucleotides were synthesised by Eurogenetec and Sigma-Genosys and purified by PAGE/HPLC. Murine recombinant Flt3-L was from Peprotech. Murine *Ifna1* and *Il6* qRT-PCR primers were from RealTimePrimers.com. LL-37 (LLGDFFRKSKEKIGKEFKRIVQRIKDFLRNLVPRTES) and scrambled LL-37: RSLEGTDRFPFVRLKNSRKLEFKDIKGIKREQFVKIL) were custom synthesised by Almac (East Lothian, Scotland) using Fmoc solid phase synthesis and reversed phase HPLC purification. For each peptide, identity was confirmed by electrospray mass spectrometry, purity (>95% area) by RP-HPLC and net peptide content determined by amino acid analysis.

### Antibodies

Flow cytometry antibodies: B220/CD45R-e450, CD3-e780, CD11b-APC, CD11c-e780, CD19-e780, CD49b-e780 CD86-Alexa Fluor 488, NK1.1-e780 from eBioscience. CD8α-PECF594, CD40-PE from BD Pharmingen. B220-BV650, CD11b-BV711, CD11c-BV421, CD40-FITC, CD80-BV605, CD86-A700, F4/80-PE-Cy7, Ly6C-BV570, Ly6G-APC-Cy7, MHC II-PerCP-Cy5.5 from Biolegend. PDCA-1-PE, FcR block from Miltenyi Biotec. The S9.6 monoclonal antibody against RNA:DNA hybrids was purified from hybridoma cell line HB-8730 (ATCC-LGC Promochem) supernatant using a Protein A/G column as previously described (Pohjoismaki *et al*, 2010).

### Generation and purification of RNA:DNA hybrids

PAGE/HPLC-purified RNA and DNA oligonucleotides were reconstituted to 100 μM with nuclease-free distilled water (Invitrogen). RNA:DNA hybrids were formed using 20 μM of each complementary oligonucleotide denatured in 60 mM KCl, 50 mM Tris (pH 8.0) at 95°C for 5 min followed by gradual cooling to room temperature. Resulting RNA:DNA duplexes were concentrated by precipitation with 2.5 volumes of 100% ethanol, 0.3 M sodium acetate (pH 5.2) and resuspended in an appropriate volume of nuclease-free TE (10 mM Tris, 0.1 mM EDTA). Purification was performed on an ÄKTA FPLC™ machine (GE Healthcare) at 4°C by injecting 100 μl volume of nucleic acids onto a 24-ml Superdex 10/300 GL column (GE Healthcare) pre-equilibrated with two column volumes of nuclease-free TE. Elution was performed with equilibration buffer (TE) at a flow rate of 0.4 ml/min, with 100 μl fractions collected into sterile nuclease-free 96-well 2-ml boxes (Greiner Bio-One). Absorbance at 280 nm was recorded for each fraction. Relevant fractions containing the purified hybrid were pooled, quantified and concentrated by ethanol precipitation and analysed by native PAGE.

### Characterisation of RNA:DNA hybrids by PAGE and immunoblotting

Size separation of nucleic acids under native conditions was performed using 15% native PAGE gels, pre-run for 1 h at 5 W 4°C, samples were loaded in an equal volume of native loading buffer (30% (v/v) glycerol, 80 mM HEPES-KOH (pH 7.9), 100 mM KCl, 2 mM magnesium acetate) and electrophoresed in 1× TBE at 5 W 4°C in a vertical gel tank (EV200, Cambridge Electrophoresis). Nucleic acids were visualised using Sybr Gold (Invitrogen) according to manufacturer's instructions and imaged on a phosphorimager (FLA-2000, Fujifilm) or UV transilluminator (BioDoc-It System, UVP). Immunoblot detection of RNA:DNA hybrids was performed by transfer of electrophoresed nucleic acids to Hybond-N+ (GE Healthcare) using a Trans-Blot® Semi-Dry Electrophoretic Transfer Cell (Bio-Rad Laboratories), in 1× TBE for 30 min at a maximum current of 3 mA/cm^2^. Nucleic acids were cross-linked to the membrane by exposure to 1200 mJ/cm^2^ UV (Stratalinker, Stratagene). Membrane was pre-blocked overnight in 1% (w/v) blocking agent (GE Healthcare) in 0.1% (v/v) Tween-20/PBS at 4°C, washed in 0.1% (v/v) Tween-20/PBS, probed with 200 ng/ml S9.6 antibody in 2% (w/v) BSA/PBS overnight at 4°C, and then incubated with HRP-conjugated goat anti-mouse IgG antibody (1:5,000, Dako Corporation) for 1 h at room temperature, followed by ECL detection (GE Healthcare).

### Preparation of bone marrow-derived Flt3-L dendritic cells

All mice used were on a C57BL/6J background, male and aged 8–14 weeks. Bone marrow was harvested from femurs and tibiae and red blood cells lysed with 0.168 M NH_4_Cl (pH 7.2) before culture in RPMI 1640 (Sigma Aldrich) supplemented with 10% (v/v) heat inactivated FCS, 2 mM l-glutamine, 50 μM β-mercaptoethanol, 100 U/ml penicillin, 100 μg/ml streptomycin and 200 ng/ml recombinant murine Flt3-L for 8 days. DCs were harvested by gentle rinsing with culture medium.

### Isolation of human PBMCs

PBMCs were isolated from peripheral blood from adult donors using Lymphoprep (Stemcell Technologies), according to the manufacturer's instructions.

### Cell stimulation with nucleic acids

Cells were seeded at 1 × 10^6^ cells into 48-well plates in culture medium without antibiotics and transfected with nucleic acids using Lipofectamine LTX (Invitrogen) and OptiMEM (Invitrogen) according to manufacturer's instructions. 1 μg/ml of R:D60, R:D45 or poly U were used for transfection or 1 μg/ml CpG-ODN added to the culture medium in a final volume of 500 μl/well and cells incubated at 37°C for 18 h. Alternatively, 1 μg/ml R:D45 was complexed with 25 μg/ml LL-37 or scrambled LL-37 peptide, before cell incubation as above. Supernatants for ELISAs were harvested, centrifuged at 1500 *g* for 5 min at 4°C and stored at −80°C. Cells were stained for analysis by flow cytometry or analysis of gene expression by qRT-PCR.

### Flow cytometry

Samples were acquired using FACS LSR II and FACS Canto II using BD FACSDiva software and analyzed with FlowJo v.9 software (Tree Star). FLDC subsets were sorted using a FACS Aria II (BD Biosciences) following staining with CD11c, B220 and PDCA-1 antibodies. Post-FACS sorting purity of each population was > 95%.

### ELISAs

Murine IL-6 and TNF-α and human IL-6 were quantified using Duoset Kits (R&D Systems) and human IFN-α using an IFN-α pan specific ELISA kit (Mabtech). Murine IFN-α was determined using 96-well microtitre plates (Nunc) coated with monoclonal rat anti-mouse IFN-α (clone RMMA-1, PBL Interferon Source) at 910 ng/ml overnight at room temperature. After blocking with 5% (w/v) BSA/PBS for 1 h at room temperature, 50 μl of supernatant sample was added overnight at 4°C, and detected with polyclonal rabbit anti-mouse IFN-α (PBL Interferon Source) at 80 ng/ml for 2 h, HRP-conjugated donkey anti-rabbit (Jackson ImmunoResearch Laboratories) at 80 ng/ml for 1 h, and BM Blue POD substrate (Roche). Recombinant mouse IFN-α3 (PBL Interferon Source) was included as a standard.

### Analysis of gene expression by qRT-PCR

RNA was extracted from cells using an RNeasy Mini kit (Qiagen) with on-column DNase I treatment. For 1^st^ strand cDNA synthesis, 1 μg RNA, 40 U Protector RNase Inhibitor (Roche), 100 pmol random primers (Promega), 5 mM RNase-free DTT in 14 μl was incubated at 70°C for 5 min, cooled on ice for 5 min before the addition of 1 μM dNTPs (Invitrogen), 20 U AMV Reverse Transcriptase (Roche), 1× AMV Reverse Transcriptase buffer in 20 μl with incubation at 42°C for 1 h, 75°C for 8 min. For qRT-PCR, reactions containing 1 μl of cDNA, 1× Brilliant II Sybr Green qPCR Master Mix (Stratagene), 0.3 μM passive reference dye (ROX) and 0.2 μM of each primer in a 10 μl volume were amplified in an ABI Prism HT7900 Sequence Detection System (Applied Biosciences), for 2 min at 50°C, 10 min at 95°C followed by 40 cycles of 15 s at 95°C, 1 min at 60°C. Expression of each target gene was normalised to *Actb* (β-actin) using the comparative *C*_T_ method (Livak & Schmittgen, 2001).

### Immunofluoresence

Day 8 FLDCs from C57BL/6 mice were transfected with 1 μg/ml fluorescently labelled R:D45 or the fluorescently labelled non-complementary oligonucleotides ssRNA45 and ssDNA60 using Lipofectamine LTX. After 1 h, cells were adhered to polysine slides (Thermo Scientific), fixed with 4% PFA and nuclei stained with DAPI. To stain endolysosomal compartments, 2 μM LysoTracker Green DND-26 (Life Technologies) was added to the cells for 1 h before fixation. To visualise RNA:DNA hybrids using the S9.6 antibody, fixed cells were permeabilised using 0.2% (v/v) Triton X-100/PBS for 2 min, blocked for 1 h with 2% (w/v) BSA/PBS and incubated with 1 μg/ml S9.6 antibody, which was detected using 1:500 dilution FITC-conjugated goat anti-mouse IgG (Molecular Probes). Images were taken on a Nikon A1R confocal microscope comprising of a Nikon Eclipse TiE inverted microscope with Perfect Focus System. Image capture was performed using Nikon Nis-Elements C (Nikon Instruments Europe). 3 channel images were acquired by consecutive scanning with only one laser line active per scan.

### *In vivo* studies of RNA:DNA hybrid response

Female age-matched C57BL/6J mice received a 200 μl intraperitoneal injection of PBS alone, 80 μg R:D45 diluted in PBS or 80 μg R:D45 (or sterile water as a control) complexed to Invivofectamine as per manufacturer's instructions and further diluted to the required concentration in PBS. 12 h post-injection, spleens were harvested and serum samples were collected. Spleens were digested at 37°C (with tilting and shaking) for 20 min with 0.4 U/ml Liberase TL (Roche) and 80 U/ml DNase I type IV (Sigma-Aldrich). Single-cell suspensions were prepared by mechanically disruption through a 70-μm cell strainer. Red blood cell lysis was performed on the single-cell suspension and these cells prepared for flow analysis of phenotypic activation of DC populations. Dead cells were excluded by staining with LIVE/DEAD Fixable Aqua Dead Cell Stain (Invitrogen). Live-singlet lineage-negative (CD3, CD19, CD49b, Ly6G, NK1.1) CD11c^hi^ B220^−^ cells were gated as cDCs, whilst CD11c^mid^ B220^+^ PDCA-1^+^ Ly6C^+^ cells were defined as pDCs. Inflammatory monocytes (CD11b^+^, Ly6C^+^) and macrophages (CD11b^+^, F4/80^hi^) were excluded from this analysis.

### Expression and purification of truncated TLR9 ectodomain

The truncated C-terminal ectodomain fragment of mouse TLR9 (mTLR9-cECD) was expressed in a baculovirus-insect cell system and purified as described previously (Li *et al*, 2012). Briefly, a gene encoding the proteolytically processed fragment (residues 474–824) mTLR9-cECD with an N-terminal eight-histidine purification tag, followed by the linker sequence Ser-Ser-Gly and a tobacco etch virus protease cleavage site, was cloned into the pAcGP67-A vector (BD Biosciences) in frame with the baculovirus gp67 signal sequence. mTLR9-cECD was expressed in Tni insect cells (Expression Systems). Cells were infected with 1% (v/v) of third-passage baculovirus stock. After culture in suspension for 72 h at 27°C mTLR9-cECD was extracted from intracellular compartments with 50 mM Tris pH 7.5, 500 mM NaCl, 10% glycerol, 5 mM β-mercaptoethanol, 1% Fos-Choline 12. mTLR9-cECD was purified by nickel affinity chromatography with a HisTrap HP column (GE Healthcare), followed by cation exchange chromatography with a MonoS column (GE Healthcare) and size-exclusion chromatography with a Superdex 200 10/300 GL column (GE Healthcare). The size-exclusion buffer was 20 mM MES pH 5.6, 100 mM NaCl, 2 mM β-mercaptoethanol.

### Fluorescence anisotropy polarization binding measurements

To determine the binding affinities of R:D60, ssRNA60 and ssDNA60, mTLR9-cECD was titrated from a 0.2 mM stock solution into a 0.2 μM solution of 3′ Cy3-labeled R:D60 for final mTLR9-cECD concentrations between 10 nM and 3.0 μM in 20 mM MES pH 5.6, 100 mM NaCl, 2 mM β-mercaptoethanol. Binding studies were performed at 25°C. The fluorescence anisotropy depolarization was recorded after 10 min equilibration with a PTI Quantamaster C-61 two-channel fluorescence spectrophotometer. The TAMRA fluorophore was excited at 550 nm and the emission was recorded at 570 nm, with a 6-nm slit width. Fluorescence anisotropy was determined as described (Grove *et al*, 2010). The binding constant of mTLR9-cECD was determined with Origin 7 (OriginLab) by fitting the data to equation 1 (Inglese *et al*, 1989):


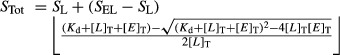
(1)

where *S*_Tot_ is the signal (fluorescence intensity or fluorescence anisotropy) of ODN at protein concentration [*E*]_T_; *S*_L_ is the signal of labeled ODN, L; *S*_EL_ is the signal of ODN in the plateau region of the binding curve; [*L*]_T_ is the total concentration of ODN added; *K*_d_ is the dissociation constant. The data were initially fit to three parameters [*L*]_T_, *S*_EL_ and *K*_d_ using the measured values for *S*_L_, *S*_Tol_ and [*E*]_T_. The overall fit was selected such that the values for [*L*]_T_ and *S*_EL_ coincided with the measured values for these parameters.

### Detection of MMLV RNA:DNA hybrids

B3T3 mouse fibroblast cells were transfected with pNCS (Yueh & Goff, 2003) (kindly provided by George Kassiotis) and passaged 12–15 times to allow the cells to accumulate a high viral load of Moloney Murine Leukemia Virus (MMLV, encoded by pNCS). Untransfected control cells were cultured in parallel. Cytoplasmic nucleic acids (NA) were extracted from cells by resuspending in 1 ml of cytoplasmic buffer (20 mM Tris–HCl pH 7.5, 10 mM KCl, 10 mM EDTA, 10% (w/v) glycerol, 0.1% (v/v) Triton X-100) and incubating on ice for 5 min. Nuclei were removed by 15 min centrifugation at 1,000 *g* 4°C, and heavy organelles by subsequent centrifugation for 15 min at 10,000 *g* 4°C. NA were extracted from the remaining supernatant by the addition of 0.5% SDS followed by two extractions with phenol:chloroform:isoamylalcohol (25:24:1) and one with chloroform, and precipitated with an equal volume of isopropanol. NA was similarly isolated from cytoplasmic (post-nuclear supernatants, PNS) and endosomal fractions (see below). RNA:DNA hybrids were immunoprecipitated using the S9.6 antibody. Approximately 16 μg of S9.6 antibody was incubated with 30 μl of Dynabeads Protein G (Life Technologies) and washed before incubation in 10 mM EDTA/PBS for 3 h at 4°C with 2 μg of cytoplasmic or endosomal NA. As a proof of principle control to show that S9.6 pulls down RNA:DNA hybrids from solution, 10 pmol of R:D45 was used (Supplementary Fig S6). In further control reactions, NA were incubated with Dynabeads Protein G only (i.e. no S9.6) or treated with 10 U RNase H (NEB) for 1 h at 37°C before incubation with Dynabeads Protein G (with bound S9.6). After thorough washing, bound NA were eluted in 0.6% SDS/10 mM EDTA, and both unbound and bound fractions were ethanol precipitated. MMLV DNA was subsequently detected using PCR and two different primer sets (MMLV-1 and MMLV-2, Supplementary Table S1) amplifying 75-and 104-bp fragments of the viral genome, respectively. For standard PCR FastStart PCR Master (Roche) was used for 10 μl reactions (5 min 95°C, then 35 cycles of 30 s 95°C, 30 s 60°C, 10 s 72°C) with 0.5 μl of input NA. For qPCR, 10 μl reactions containing 1 μl of input NA, 1× Brilliant II Sybr Green qPCR Master Mix (Stratagene), 0.3 μM passive reference dye (ROX) and 0.2 μM of each primer were amplified in an ABI Prism HT7900 Sequence Detection System (Applied Biosciences), for 2 min at 50°C, 10 min at 95°C followed by 40 cycles of 15 s at 95°C, 1 min at 60°C. Relative levels were calculated using the comparative *C*_T_ method (Livak & Schmittgen, 2001).

### Endosome isolation

Endosomal isolations (mixture of early and late endosomes) were performed by density gradient centrifugation as previously described, with minor modifications (de Araujo *et al*, 2008). Solutions were prepared as described above, but 10 mM EDTA was used and no protease or phosphatase inhibitors. Between 4 and 8 T75 flasks of MMLV infected B3T3 were collected and lysed using hypotonic shock in homogenisation buffer A followed by mechanical disruption by passing the cell suspension through a 23-G needle six times. The post-nuclear supernatant (PNS) was brought up to 42% sucrose and used in discontinuous sucrose gradient centrifugation (42%, 35% and 8%) in SW41 tubes for 3 h at 210,000 *g* 4°C. Endosomes were collected in 0.5–1 ml from the interface between 35% and 8% sucrose layers, and used for Western blotting and NA isolation. For Western blotting, 10 μg of total PNS protein and 30 μl of the endosomal fraction were separated on 4–12% NuPAGE (Life Technologies), transferred to PVDF membrane, blocked in 1× TBST with 5% milk and probed with anti-Rab5 antibody (Cell Signaling, 1:1,000 rabbit monoclonal C8B1) and anti-GAPDH (Abcam, 1:1,000 HRP-conjugated ab39385) respectively.

### Statistical testing

Unpaired Student's *t*-tests were performed unless otherwise stated, calculated using GraphPad Prism Software version 6.0 (La Jolla, USA). Significance levels are denoted in figures as: *P ≤ 0.05, **P ≤ 0.01, ***P ≤ 0.001, ns not significant.

## Supplementary information

for this article is available online: http://emboj.embopress.org
