# Supplementary material for: RNA:DNA hybrids are a novel molecular pattern sensed by TLR9
Source: EMBO J. 2014 Feb 21;33(6):542–58. doi: 10.1002/embj.201386117 (PMC3989650; doi:10.1002/embj.201386117)
Supplement: Supplementary file 1 [file embj0033-0542-sd1.pdf]

THE EMBO JOURNAL

Manuscript EMBO-2013-86117

# RNA:DNA hybrids are a novel molecular pattern sensed by TLR9

Rachel E Rigby, Lauren M Webb, Karen J MacKenzie, Yue Li, Andrea Leitch, Martin AM Reijns, Rachel J Lundie, Ailsa Revuelta, Donald J Davidson, Sandra Diebold, Yorgo Modis, Andrew S MacDonald and Andrew P Jackson

*Corresponding authors: Andrew Jackson, Institute of Genetics and Molecular Medicine and Andrew MacDonald, Institute of Immunology & Infection Research, University of Edinburgh*

**Review timeline:**

| | |
|---|---|
| Submission date: | 07 July 2013 |
| Editorial Decision: | 26 August 2013 |
| Revision received: | 24 November 2013 |
| Accepted: | 27 November 2013 |

Editor: Karin Dumstrei

## Transaction Report:

(Note: With the exception of the correction of typographical or spelling errors that could be a source of ambiguity, letters and reports are not edited. The original formatting of letters and referee reports may not be reflected in this compilation.)

1st Editorial Decision 26 August 2013

Thank you for submitting your manuscript to The EMBO Journal. I am very sorry for the delay in getting back to you with a decision, but due to vacation time etc the referees requested additional time to do the review, which I granted. I have now heard back from the three referees.

As you can see below, the referees find your analysis exciting, well done and suitable for publication here. I would therefore like to invite you to submit a suitable revised version. Referees #2 and 3 raise the question if TLR9 detects DNA:RNA hybrids in the endosomes in the normal course of a viral infection. They suggest a number of ways to address this question, but there might be other possibilities as well. Adding experimental data to address this point would clearly strengthen the paper, if this becomes technical challenging or too time consuming do get in touch with me so that we can discuss this issue further.

When preparing your letter of response to the referees' comments, please bear in mind that this will form part of the Review Process File, and will therefore be available online to the community. For more details on our Transparent Editorial Process, please visit our website: http://www.nature.com/emboj/about/process.html

We generally allow three months as standard revision time. As a matter of policy, competing manuscripts published during this period will not negatively impact on our assessment of the conceptual advance presented by your study. However, we request that you contact the editor as soon as possible upon publication of any related work, to discuss how to proceed. Should you

foresee a problem in meeting this three-month deadline, please let us know in advance and we may be able to grant an extension.

Thank you for the opportunity to consider your work for publication. I look forward to your revision.

REFEREE REPORTS

Referee #1

Jackson et. al. provide compelling evidence that TLR9 (known to best recognise thioated CpG-DNA) also efficiently sense RNA:DNA hybrids containing viral sequences.They conclude that "hybrids" represent novel molecular patterns sensed by innate immune cells.
The experimental demonstration that TLR9 senses MyD88 dependent highly purified RNA:DNA hybrids is well well executed. At surprise mTLR9-cECD is shown to bind "hybrids" with unusual high affinity (Fig 7A). far higher than that of classical TLR9 ligands (see Li et. al, 2012) or ss.DNA60 (as shown here). From this point of view "hybrids" appear as better TLR9 ligands than "ss.CpG motif containing" or "non containing" DNA sequences ( not really discussed.)
One concern relates to the data detailed on page 10. Given that thioated ss. CpG DNA efficiently translocates to endosomes (where TLR9 is expressed) while natural di-ester DNA does not yet upon aided translocation stimulate TLR9 rather sequence indepently (see Haas et al, 2008,cited),it surprises that transfected ss. DNA-60 poorly stimulates Il-6 and type1 IFN secretion (see Fig. 6B). Does this imply that only "hybrids" display full TLR9 stimulatory activity ? (not really discussed). Another related option is that that higher concentrations of transfected ss.DNA-60 are needed - yet no titration data are offered. The authors need to madress/discuss these issues.Nevertheless and as it stands, I add my congratulation to a nice piece of work.

Referee #2

In this work Rigby et al present data o RNA:DNA hybrids being a PAMP stimulating innate immune responses in pDCs via TLR9. The question under investigation is interesting and relevant given this nucleic acid form being produced during several infections and also to be believed to accumulated to abnormally high levels during some autoinflammatory conditions. Although the experiments are well designed and presented, important data are still lacking in order to provide physiological relevance and to fully support the conclusions drawn by the authors.

MAJOR POINTS
1. Confocal microscopy (using the S9.6 antibody) should be used to demonstrate that the hybrid actually stays intact inside the cells.

2. Confocal microscopy should also be employed to demonstrate localization of the RNA:DNA hybrid in endosomes and the recruitment of TLR9 and MyD88.

3. Does the RNA:DNA PAMP loose potency if the DNA is methylated?

4. Data on human should be provided, preferentially with primary human pDCs.

5. The data presented in Fig. 6 on ssDNA potentially being the TLR9 PAMP should preferentially also be done in Trex1 KO cells. The ssDNA specie is much more sensitive to Trex1-mediated degradation than the hybrid.

6. The nice data in Fig 7 should be supplied with binding data for classical dsDNA.

7. The current version of the manuscript totally lacks data on the physiological relevance of RNA-DNA as a TLR9-stimulating PAMP. The authors mention a range of pathogens that could stimulate this pathway. Data should be provided on this. As a minimum that the RNA:DNA hybrid actually accumulated during one of these infections.

MINOR POINTS
1. The text refers to Fig 2G, which cannot be found on the figure (most likely 2F, right panel).

2. Figure 1 should be moved to supplemental figures.

3. Figure 4 and 5 should be merged.

Referee #3

TLR9 is one of the most extensively studied DNA sensors known so far that stimulate the production of type-I interferons, but the majority of studies on TLR9 have relied on the use of CpG DNA, which contains phosphorothioate bonds. The relevance of this type of synthetic DNA to the sensing of natural DNA such as microbial and self DNA is questionable. In this study, Rigby et al provide strong evidence that DNA/RNA hybrids can bind to TLR9 with a high affinity and that TLR9 is essential for cytokine induction by transfected DNA/RNA hybrids. This is a very well executed study and the paper is recommended for publication in a high profile journal such as EMBO J. The following comments are quite minor but attention to these points may further improve the paper.

1. Figure 4A and 4B: Ideally, WT mice should be included as a control.
2. Figure 5A and 5D: the authors should comment on why IFNa3 induction by R:D60 was only partially inhibited (~50%) in Tlr9 ko pDCs.
3. Ideally, the authors should test whether Tlr9 ko mice could produce IFNa or IFNb in response to transfection of DNA:RNA hybrids. They have the mice and they have shown that DNA:RNA hybrids could induce IFNa3 in mice and could upregulate CD86 in DCs (using invivofectamine, Figure 3).
4. The authors should comment on why DNA:RNA hybrids should be transfected using lipofectamine to stimulate TLR9, whereas CpG DNA can be added to culture media without transfection, which apparently enters the endosome/lysosome to stimulate TLR9. Is there direct evidence that DNA:RNA hybrids (e.g, using Cy3-labeled DNA or RNA) get into the endosome (rather than in the cytoplasm and nucleus, which is commonly observed after transfection) to stimulate TLR9? If the hybrids cannot be found in the endosome or lysosome, it would be difficult to understand how they stimulate TLR9 because of the receptor topology.
5. Page 13, line 3 from the bottom: "with a much higher Kd than..." should be changed to "with a much lower Kd than..."

1st Revision - authors' response 24 November 2013

**<u>Point by Point Responses to the Referees</u>**

We are grateful for the supportive and encouraging comments from the referees. As advised by the editor, we have focussed our additional experimentation on improving physiological relevance of RNA:DNA hybrid detection and their localisation to endosome. In particular based on suggestions from reviewer 2 (points 1,2, 7) and reviewer 3 (point 4), we are now able to provide evidence that:-

1. RNA:DNA hybrids remain intact when transfected into dendritic cells (Figure 6).

2. Transfected RNA:DNA hybrids can localise to endolysosomes in dendritic cells (Figure 6).

3. Viral RNA:DNA hybrids are detectable in endosomal fractions of cells infected with the retrovirus MMLV (Figure 8).

Taken together, these experiments support the notion that TLR9 sensing of RNA:DNA hybrids is endosomal and has physiologically relevant.

We also demonstrate, as suggested by the reviewers, that induction of a cytokine response by RNA:DNA hybrids is not species-specific, by *ex vivo* analysis of human PBMCs.

We considered if there were additional ways to demonstrate physiological relevance, but concluded that a more comprehensive demonstration of physiological sensing of hybrids during a viral infection lies beyond the scope and timeframe of this current study. The central issue here is that multiple nucleic acid species are simultaneously generated during an active viral infection. Therefore designing experimental systems to precisely define the individual roles of all naturally occurring nucleic acid species (DNA, RNA and RNA:DNA hybrids) within a cell will be challenging, particularly as we have established here that different types of nucleic acid can be sensed by the same pattern recognition receptor.

Our point by point responses are outlined below:-

**Referee #1**

*Jackson et. al. provide compelling evidence that TLR9 (known to best recognise thioated CpG-DNA) also efficiently sense RNA:DNA hybrids containing viral sequences. They conclude that "hybrids" represent novel molecular patterns sensed by innate immune cells. The experimental demonstration that TLR9 senses MyD88 dependent highly purified RNA:DNA hybrids is well well executed. At surprise mTLR9-cECD is shown to bind "hybrids" with unusual high affinity (Fig 7A). far higher than that of classical TLR9 ligands (see Li et. al, 2012) or ss.DNA60 (as shown here). From this point of view "hybrids" appear as better TLR9 ligands than "ss.CpG motif containing" or "non containing" DNA sequences ( not really discussed.) One concern relates to the data detailed on page 10. Given that thioated ss. CpG DNA efficiently translocates to endosomes (where TLR9 is expressed) while natural di-ester DNA does not yet upon aided translocation stimulate TLR9 rather sequence indepently (see Haas et al, 2008,cited),it surprises that transfected ss. DNA-60 poorly stimulates Il-6 and type1 IFN secretion (see Fig. 6B). Does this imply that only "hybrids" display full TLR9 stimulatory activity ? (not really discussed). Another related option is that that higher concentrations of transfected ss.DNA-60 are needed - yet no titration data are offered. The authors need to madress/discuss these issues.Nevertheless and as it stands, I add my congratulation to a nice piece of work.*

We thank the referee for his/her kind comments on the quality and relevance of our experimental work. The referee raises an interesting question regarding the relative efficacy of different TLR9 ligands. This was not something we had considered, our primary focus of our study being to establish hybrids as a novel molecular pattern. He/she is correct that the same concentration of ssDNA is much less immunostimulatory. Such a weak cytokine response to this concentration of single-stranded phosphodiester DNA is in keeping with previous studies. In the current study, ssDNA60 was used at 0.5 ug/ml which equates to a 25 nM concentration (0.025 pmol/ul). On the titration curve shown in Haas et al. 2008 (Figure 1D), PD1668 + DOTAP is weakly stimulatory when used at this concentration in Flt3-derived dendritic cells (FLDCs). Also, Yasuda et al. 2006 (Figure 2B/C wildtype PD + DOTAP) show that levels of IFN-$\alpha$ and IL-6 produced by FLDCs transfected with PD ODNs is dose dependent, with only low amounts of cytokine produced in response to < 50 nM ODN, although levels vary depending on which ODN is used. As these previous studies demonstrated single-stranded DNA is immunostimulatory at higher concentrations, rather than implying that hybrids are immunostimulatory and ssDNA is not, we believe that this indicates that TLR9 sensing is most likely a function of ligand affinity, with stronger binders able to activate the receptor at lower concentrations.

We now discuss this important point on page 16 of the discussion as follows:

**"**The RD60 RNA:DNA hybrid is notably more immunostimulatory than the corresponding ssDNA oligonucleotide at the same concentration (Figure 5A-C). A weak cytokine response at this relatively low concentration (25 nM) is in keeping with previous studies of phosphodiester ssDNA oligonucleotides, (Haas 2008, Yasuda, 2006), which demonstrated immunostimulation at higher concentrations. The differential response observed here, correlates with the higher affinity of the TLR9 receptor for the hybrid (Figure 7), therefore binding affinity may be important in determining cytokine response. Other factors such as the relative stability of ssDNA and RNA:DNA hybrids within the endosome could also play a role. Future studies correlating the relative abundance and

© European Molecular Biology Organization 

receptor affinities of naturally occurring TLR9 ligands with cytokine response may therefore be informative in defining which are most relevant to disease."

**Referee #2**

*In this work Rigby et al present data o RNA:DNA hybrids being a PAMP stimulating innate immune responses in pDCs via TLR9. The question under investigation is interesting and relevant given this nucleic acid form being produced during several infections and also to be believed to accumulated to abnormally high levels during some autoinflammatory conditions. Although the experiments are well designed and presented, important data are still lacking in order to provide physiological relevance and to fully support the conclusions drawn by the authors.*

*MAJOR POINTS*
*1. Confocal microscopy (using the S9.6 antibody) should be used to demonstrate that the hybrid actually stays intact inside the cells.*

We thank the reviewer for suggesting this nice experiment. We now provide confocal immunofluorescence data demonstrating that the anti-hybrid antibody S9.6 detects intact RNA:DNA hybrids in Flt-3 generated dendritic cells (FLDCs). Additionally, we have examined the localisation of fluorescently labelled ssRNA and ssDNA oligonucleotides. When hybridised, these completely colocalised, in keeping with these hybrids remaining intact within cells.

This new data is now included in the paper as Figure 6.

*2. Confocal microscopy should also be employed to demonstrate localization of the RNA:DNA hybrid in endosomes and the recruitment of TLR9 and MyD88.*

To address, this (and Reviewer 3, point 4) we performed colocalisation studies with fluorescently labelled RNA:DNA hybrids and LysoTracker (Life Technologies) a marker of acidified endolysosomal compartments. Colocalisation of RNA:DNA hybrids with LysoTracker was seen in FLDCs, consistent with endosomal localisation (Figure 6E), in keeping with previous studies that have demonstrated that cellular uptake of Lipofectamine-nucleic acid liposomal complexes occurs via the endosome (Molecular Therapy 9:443). Additionally, we also demonstrate that viral RNA:DNA hybrids are present in endosomal fractions during MMLV infection (Figure 8), consistent with the detection of RNA:DNA hybrids in the endosome.

We also attempted to demonstrate colocalisation with TLR9. However, this has not been possible to achieve in the timeframe available for revision. Staining of TLR9 in FLDCs with the anti-TLR9 antibody available to us was not successful and we note that many previous studies have used overexpressed tagged TLR9 for localisation studies (Barton et al. Nat Immunol. 2005, 7:49-56; Lee et al. eLife 2013;2:e00291; Latz et al. Nat. Immunol, 2004, 5:190-198), possibly due to sensitivity issues in detecting endogenous TLR9. Irrespective of this, given the well established cellular mechanisms by which TLR9 is recruited from the endoplasmic reticulum to endolysosomes, where it is proteolytically processed and binds its ligand, our demonstration of localisation of RNA:DNA hybrids to endosomes, makes it likely that TLR9/MyD88 sense hybrids in the endosome. In support of this, in our original submission we demonstrated that RNA:DNA sensing is choloroquine dependent (Figure 4I), in keeping with signalling occurring in an acidified endosomal compartment.

*3. Does the RNA:DNA PAMP loose potency if the DNA is methylated?*

We presume the reviewer has raised this question, because of the longstanding literature demonstrating that TLR9 sensing of CpG phosphorothioate ODNs is lost on methylation. However, this does not seem to be the case for phosphodiester backbone nucleic acids, according to the findings of Haas and colleagues (Immunity 2008; 28:315-323). Furthermore, RNA:DNA hybrids, generated by reverse transcription during infection (Hu and Hughes, Cold Spring Harb Perspect Med 2012; 2:a006882) would not be expected to be methylated. Therefore, whether methylation affects potency is not central to the current question of whether RNA:DNA hybrids are molecular

patterns sensed by TLR9. We agree with the reviewer that addressing this would be an interesting future experiment, but feel that this lies outwith the scope of the current study.

*4. Data on human should be provided, preferentially with primary human pDCs.*
To demonstrate that hybrid sensing is not species-specific, we have established that RNA:DNA hybrids do indeed stimulate an immune response in human cells. We now show that, R:D45 robustly induces IFN-α and IL-6 production when transfected into human *ex-vivo* PBMCs (Figure 3C).

*5. The data presented in Fig. 6 on ssDNA potentially being the TLR9 PAMP should preferentially also be done in Trex1 KO cells. The ssDNA specie is much more sensitive to Trex1-mediated degradation than the hybrid.*

The purpose of this figure (now Figure 5) is to demonstrate that an intact RNA:DNA hybrid rather than the ssDNA component ssDNA60 is being detected in wild-type cells. ssDNA is included in this experiment to demonstrate that if hybrid dissociation were to happen (or if RNase H mediated hydrolysis occured) generating ssDNA, then this would not be itself sufficiently immunostimulatory to account for the response seen. The proposed degradation of ssDNA by Trex1 would in fact therefore support our argument that intact hybrid rather than ssDNA accounts for the cytokine response seen.

As pointed out by reviewer 1, the likely explanation for the low ssDNA response is its lower receptor affinity, resulting in reduced activation at the low concentration used (chosen so that it was equimolar with the hybrid). Furthermore, as Lipofectamine-nucleic acid uptake is endosomally-mediated (Molecular Therapy 9:443), it will not necessarily be exposed to the Trex1 exonuclease, which localises to the cytosolic side of the endoplasmic reticulum.

Therefore, although it might in part explain why ssDNA is less immunostimulatory, we do not believe that repeating this experiment in *Trex1*$^{-/-}$ cells will significantly enhance evidence for RNA:DNA hybrids remaining intact in cells.

*6. The nice data in Fig 7 should be supplied with binding data for classical dsDNA.*

We thank for reviewer for his/her appreciation of this data. In fact, binding data for classical dsDNA has been previously generated by us (Yorgo Mordis and Yue Li, Embo Journal, 2012). Given, that data on the binding affinity of dsDNA is already published, we have not added it to the figure. Furthermore the purpose of Figure 7 is to provide biophysical evidence to support the binding of RD60 to the TLR9 receptor, rather than its single-stranded components, and so binding data on dsDNA would not inform further on this result. However, given the reviewer's comment, we now make reference to binding data for dsDNA on page 13, "This was also stronger than a previously tested dsDNA ligand (Li et al, 2012)".

*7. The current version of the manuscript totally lacks data on the physiological relevance of RNA-DNA as a TLR9-stimulating PAMP. The authors mention a range of pathogens that could stimulate this pathway. Data should be provided on this. As a minimum that the RNA:DNA hybrid actually accumulated during one of these infections.*

We thank the reviewer for encouraging us to pursue this possibility further, and we now provide demonstration that RNA:DNA hybrids are detectable in cytoplasmic and endosomal fractions of cells infected with the retrovirus MMLV. These additional findings are provided as Figure 8, and we include the following text in the results section, documenting these findings:-

"Many pathogens, most notably retroviruses, generate RNA:DNA hybrids as replication intermediates within an infected cell. To establish if significant levels of intact RNA:DNA hybrids were present within infected cells, we used the S9.6 antibody to affinity-purify RNA:DNA hybrids from B3T3 fibroblasts infected with the retrovirus Moloney Murine Leukaemia Virus (MMLV). Following S9.6 pull down of RNA:DNA hybrids from cytoplasmic extracts of infected cells, viral nucleic acid was detectable by PCR using virus-specific primers (Figure 8A, B). As PCR detects both MMLV DNA and RNA:DNA hybrids, the specificity of the S9.6 pulldown for RNA:DNA hybrids was confirmed by pre-treatment with RNase H, which abrogated the PCR signal, consistent

© European Molecular Biology Organization

with pull down of intact RNA:DNA hybrids by the S9.6 antibody. Quantification by qPCR using two different sets of primers showed that S9.6 immunoprecipitates 4.1 + 1.1% of MMLV cytoplasmic DNA (Figure 8B, $p \leq 0.03$), demonstrating that significant levels of intact RNA:DNA hybrids can accumulate during viral infection. As detection of TLR9 ligands occurs in endosomes, we next sought to determine if viral RNA:DNA hybrids could be detected in the endosomes of infected cells. Endosomal fractions were prepared from B3T3 cells infected with MMLV by discontinuous sucrose gradient ultra-centrifugation and validated by immunoblotting to confirm the presence of the endosome marker Rab5, and absence of GAPDH, a cytosolic enzyme, from these fractions (Figure 8C). Subsequently, the S9.6 antibody was used to pull down RNase H-sensitive nucleic acids from this endosomal fraction (Figure 8D), consistent with the presence of viral RNA:DNA hybrids in the endosomes of MMLV infected cells. "

*MINOR POINTS*
*1. The text refers to Fig 2G, which cannot be found on the figure (most likely 2F, right panel).*

We apologise for this error. The correct labelling was indeed 2F, right panel. We have rectified this in the revised manuscript (now Fig 2E, to follow order of figure panels in text).

*2. Figure 1 should be moved to supplemental figures.*

Biochemical characterisation of the RNA:DNA hybrids and their purification, underpin subsequent immunological experiments. As outlined in paragraph 2 of the discussion, precise definition of substrates has been central to understanding pattern recognition receptor-ligand interactions. Given that we are within the figure limit permitted in Embo Journal (9 figures), we therefore would prefer to retain this data as Figure 1.

*3. Figure 4 and 5 should be merged.*
We have merged Figure 4 and 5 as suggested.

**Referee #3**

*TLR9 is one of the most extensively studied DNA sensors known so far that stimulate the production of type-I interferons, but the majority of studies on TLR9 have relied on the use of CpG DNA, which contains phosphorothioate bonds. The relevance of this type of synthetic DNA to the sensing of natural DNA such as microbial and self DNA is questionable. In this study, Rigby et al provide strong evidence that DNA/RNA hybrids can bind to TLR9 with a high affinity and that TLR9 is essential for cytokine induction by transfected DNA/RNA hybrids. This is a very well executed study and the paper is recommended for publication in a high profile journal such as EMBO J. The following comments are quite minor but attention to these points may further improve the paper.*

We thank the reviewer for his/her comments on the importance, execution and relevance of our study.

1. *Figure 4A and 4B: Ideally, WT mice should be included as a control.*

WT mice were included as controls in this experiment, and we presented the data represented as percentage cytokine produced normalised to C57BL/6 control mice, as indicated on the axis. This allowed us to correct for inter-experimental variation in cytokine levels, and present data from all 3 experiments in one graph, rather than showing a representative experiment. We have revised the figure legend to make this clearer, now stating that WT mice were included as controls in this experiment on page 22.

*"The cytokine response to R:D60 is absent in Myd88-/-Trif-/- but not Ips-1-/- mice. FLDCs derived from MyD88-/-;Trif-/- , Ips-1-/- and wild-type (C57BL/6) control mice were transfected with R:D60. Supernatant levels of IL-6 and IFN-α are represented as percentage of cytokine produced by C57BL/6 wild-type controls included in each experiment."*

*2. Figure 5A and 5D: the authors should comment on why IFNa3 induction by R:D60 was only partially inhibited (~50%) in Tlr9 ko pDCs.*

We agree with the reviewer that this is in an interesting observation, though a residual response was not apparent in the R:D45 experiment (Figure 4G). We discussed this briefly in the results section of the original submission, but given the reviewer's comment now expand on this to say: –

"Residual IFN-α secretion by Tlr9-/- pDCs in response to R:D60 could suggest an additional hybrid sensing receptor in this cell type, in which case, DHX9 or DHX36 could be plausible candidates given that they have been reported to be Myd88 dependent sensors (Kim et al. 2010)."

*3. Ideally, the authors should test whether Tlr9 ko mice could produce IFNa or IFNb in response to transfection of DNA:RNA hybrids. They have the mice and they have shown that DNA:RNA hybrids could induce IFNa3 in mice and could upregulate CD86 in DCs (using invivofectamine, Figure 3).*

We agree with the reviewer that it would be very interesting to undertake further experiments directly addressing the role of TLR9 in sensing RNA:DNA *in vivo*. Importantly, the lab in which the experiment shown in Figure 3 of the original submission was performed (A. MacDonald) has just relocated to Manchester from Edinburgh, with considerable disruption and downtime. For practical and unavoidable reasons, it has therefore not been possible to perform an additional set of *in vivo* experiments during the period of time available to us for revision. Although we have clearly establish that hybrids are sensed in a TLR9 dependent manner in FLDCs *in vitro*, the observation that there is residual interferon secretion in TLR9-/- pDCs (point 2) suggests that other sensors could exist. *In vivo* redundancy in hybrid sensing, as a result of additional sensors in other cell types, would therefore be a possibility, particularly given the plethora of receptors now reported to sense DNA and RNA. Therefore, though we would expect the experiment suggested by the reviewer to demonstrate that TLR9 is the dominant RNA/DNA sensor *in vivo*, a negative result would by no means rule out TLR9 as such a sensor. We suggest that such an experiment would nicely form the basis of future work to determine if additional hybrid sensors collaborate *in vivo*.

*4. The authors should comment on why DNA:RNA hybrids should be transfected using lipofectamine to stimulate TLR9, whereas CpG DNA can be added to culture media without transfection, which apparently enters the endosome/lysosome to stimulate TLR9.*

CpG-A DNA contains poly G tails, resulting in substantial self aggregation, which presumably accounts for its ability to be taken up from the culture media without transfection. It is therefore unusual, as nucleic acids generally require additional assistance (transfection/electroporation) to be delivered into the cell.
To address this point in the text, we have added the following to the revised manuscript (page 21)

"CpG A could be added to cultures without complexing to Lipofectamine, as it generates large macromolecular aggregates due tounusual self aggregating properties, sufficient to stimulate spontaneous cellular uptake (Wu et al 2010)."

*Is there direct evidence that DNA:RNA hybrids (e.g, using Cy3-labeled DNA or RNA) get into the endosome (rather than in the cytoplasm and nucleus, which is commonly observed after transfection) to stimulate TLR9? If the hybrids cannot be found in the endosome or lysosome, it would be difficult to understand how they stimulate TLR9 because of the receptor topology.*

We thank the reviewer for the suggestion to investigate the intracellular location of the hybrids, and now show that transfected RNA:DNA hybrids can be seen to colocalise with endolysosomal compartments (Figure 6E), as detailed in response to Referee 2, point 2, above. This is also consistent with the existing literature as Lipofectamine has been established to deliver nucleic acids intracellularly via endocytosis. Furthermore, we are also able to show that viral hybrids are present in endosomal fractions, and therefore available for detection by TLR9 (Figure 8).

*5. Page 13, line 3 from the bottom: "with a much higher Kd than..." should be changed to "with a much lower Kd than..."*
We thank the reviewer for pointing out this error, and have now corrected this sentence.

Accepted 27 November 2013

Thank you for submitting your revised manuscript to The EMBO Journal. I asked referee #2 to take a look at the revision and as you can see below, the referee appreciates the introduced changes. I am therefore very pleased to accept the paper for publication here. You will hear back from us within the next few days regarding the licences etc.

Thank you for submitting your work to us

REFEREE REPORT

Referee #2:

This reviewer finds that the authors have dealt with most of the critics raised in a satisfactory manner, and therefore now recommends acceptance.
