# Supplementary material for: RNA:DNA hybrids are a novel molecular pattern sensed by TLR9
Source: EMBO J. 2014 Feb 21;33(6):542–58. doi: 10.1002/embj.201386117 (PMC3989650; doi:10.1002/embj.201386117)
Supplement: Supplementary file 2 [file embj0033-0542-sd2.pdf]

Figure S1

A Primary MEFs

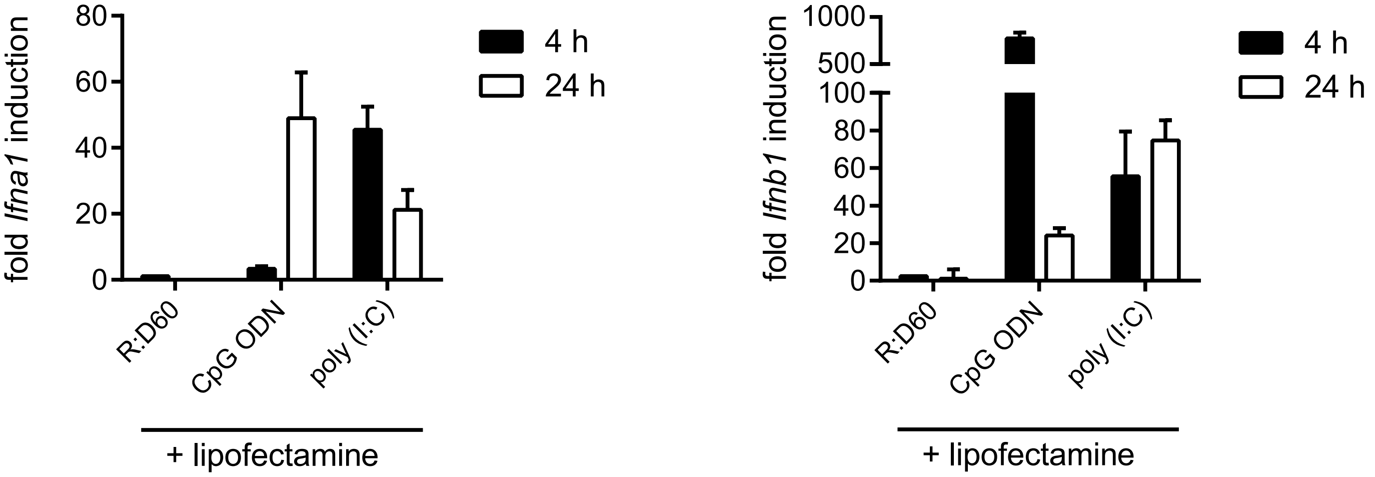

B BMDMs

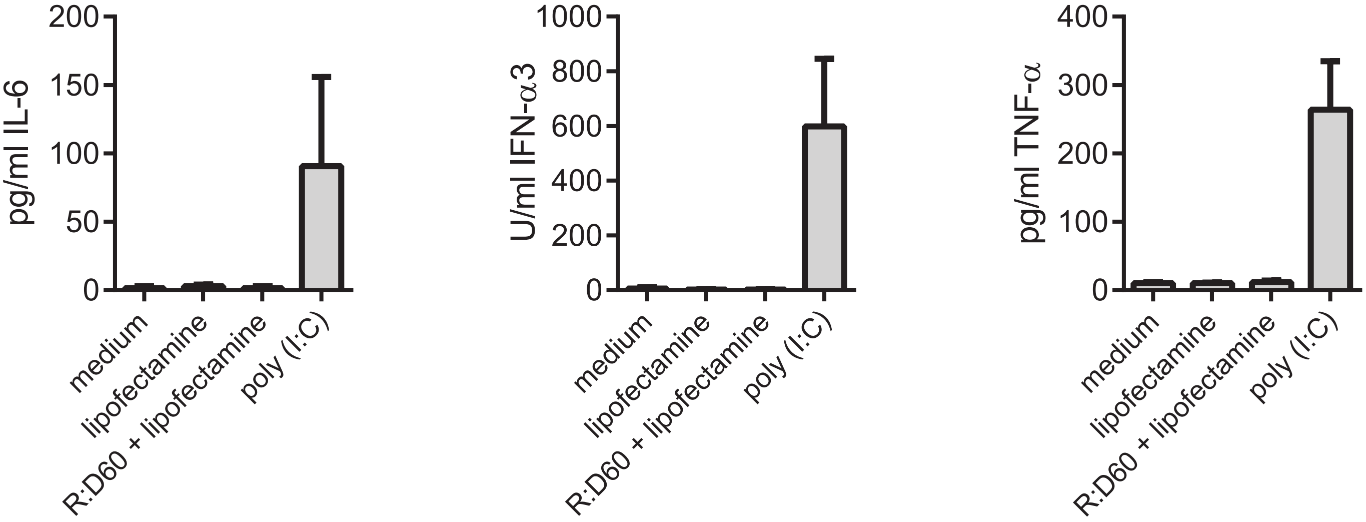

Figure S1. RNA:DNA hybrids do not induce cytokine production in primary MEFs or BMDMs

(A) Type I IFN gene expression in early passage (< 3 passages) primary murine embryonic fibroblasts (MEFs) transfected with 25 μg/ml R:D60, 5 μg/ml CpG ODN (ODN1585) or 25 μg/ml poly (I:C) using Lipofectamine 2000 was measured by qRT-PCR at the time points indicated post-transfection. Data are shown as normalised fold change relative to Lipofectamine alone, representative of three independent experiments + s.d. of n=3 replicates.

(B) Bone marrow derived macrophages (BMDMs) derived by culturing bone marrow from C57BL/6 mice in the presence of M-CSF were transfected with 1 μg/ml R:D60 complexed with Lipofectamine LTX or a control stimulated by the addition of 1 μg/ml poly (I:C) to the culture medium. Supernatant levels of the cytokines IL-6, IFN-α and TNF-α were quantified 18 h later by ELISA. Data shown are the mean of at least three independent experiments + s.e.m.
