# Supplementary material for: RNA:DNA hybrids are a novel molecular pattern sensed by TLR9
Source: EMBO J. 2014 Feb 21;33(6):542–58. doi: 10.1002/embj.201386117 (PMC3989650; doi:10.1002/embj.201386117)
Supplement: Supplementary file 3 [file embj0033-0542-sd3.pdf]

Figure S2

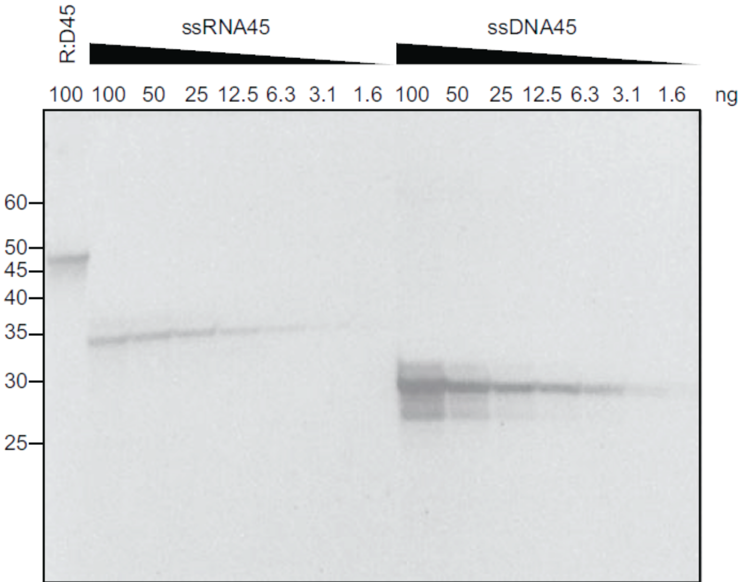
R:D45
ssRNA45
ssDNA45
100 100 50 25 12.5 6.3 3.1 1.6 100 50 25 12.5 6.3 3.1 1.6 ng
60
50
45
40
35
30
25

Figure S2. Native PAGE analysis of the R:D45 hybrid

Native PAGE analysis of a 45 bp RNA:DNA hybrid representing sequence from the HIV-1 gag gene (R:D45). 100 ng R:D45 was electrophoresed on a 15% native polyacrylamide gel with decreasing amounts of the constituent single-stranded oligonucleotides ssRNA45 and ssDNA45 to establish that a pure 45bp hybrid was formed. 200 ng of 5bp ladder (M) was included as a size control. Nucleic acids visualised with Sybr Gold.
