# Supplementary material for: RNA:DNA hybrids are a novel molecular pattern sensed by TLR9
Source: EMBO J. 2014 Feb 21;33(6):542–58. doi: 10.1002/embj.201386117 (PMC3989650; doi:10.1002/embj.201386117)
Supplement: Supplementary file 4 [file embj0033-0542-sd4.pdf]

Figure S3

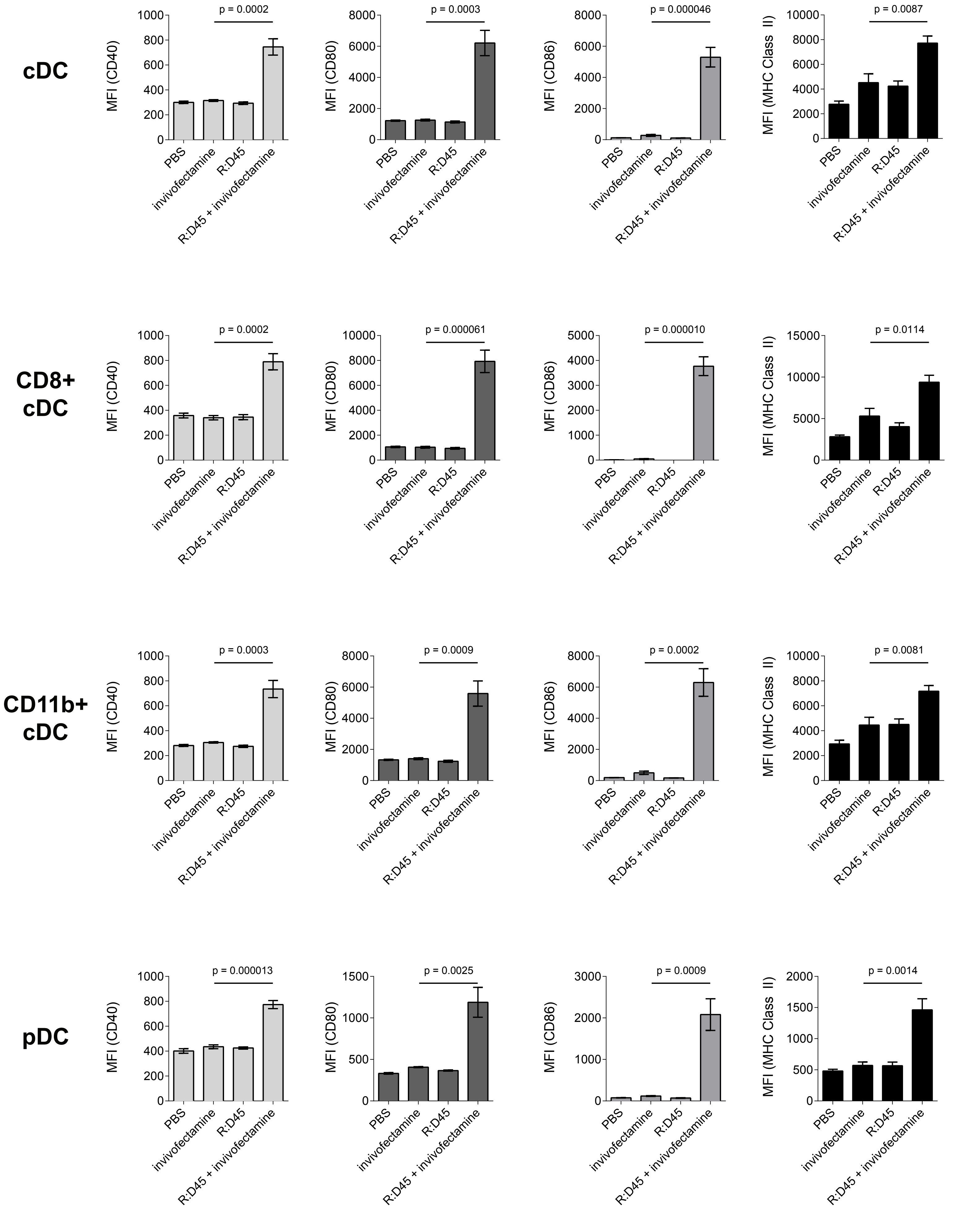

cDC
MFI (CD40)
p = 0.0002
MFI (CD80)
p = 0.0003
MFI (CD86)
p = 0.000046
MFI (MHC Class II)
p = 0.0087
CD8+ cDC
MFI (CD40)
p = 0.0002
MFI (CD80)
p = 0.000061
MFI (CD86)
p = 0.000010
MFI (MHC Class II)
p = 0.0114
CD11b+ cDC
MFI (CD40)
p = 0.0003
MFI (CD80)
p = 0.0009
MFI (CD86)
p = 0.0002
MFI (MHC Class II)
p = 0.0081
pDC
MFI (CD40)
p = 0.000013
MFI (CD80)
p = 0.0025
MFI (CD86)
p = 0.0009
MFI (MHC Class II)
p = 0.0014
PBS
invivofectamine
R:D45
R:D45 + invivofectamine

Figure S3. In vivo delivery of R:D45 using Invivofectamine is required for activation of all DC subsets

Delivery of R:D45 complexed to Invivofectamine *in vivo* phenotypically activates DCs. C57BL/6 mice were injected intraperitoneally with 80 μg R:D45 or 80 μg R:D45 complexed to Invivofectamine and the activation of splenic DC populations analysed 12 hours later by flow cytometry, as described in Figure 3. MFI values shown are from one experiment ± s.e.m. (n=5 mice per group), representative of three independent experiments.
