# Supplementary material for: RNA:DNA hybrids are a novel molecular pattern sensed by TLR9
Source: EMBO J. 2014 Feb 21;33(6):542–58. doi: 10.1002/embj.201386117 (PMC3989650; doi:10.1002/embj.201386117)
Supplement: Supplementary file 5 [file embj0033-0542-sd5.pdf]

Figure S4

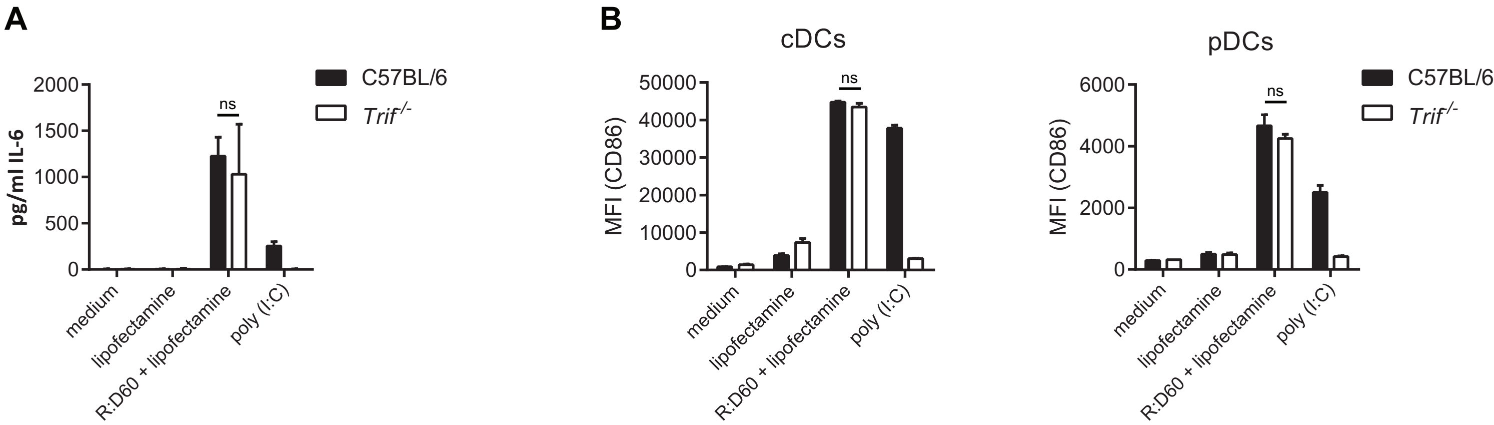

A
B
cDCs
pDCs
C57BL/6
Trif-/-
pg/ml IL-6
MFI (CD86)
ns
2000
1500
1000
500
0
50000
40000
30000
20000
10000
6000
4000
2000
medium
lipofectamine
R:D60 + lipofectamine
poly (I:C)

Figure S4. Signalling via the adaptor molecule TRIF is not required for the cytokine response to RNA:DNA hybrids in FLDCs

FLDCs derived from *Trif*$^{-/-}$ and C57BL/6 control mice were transfected with 1 μg/ml R:D60 complexed to Lipofectamine LTX or stimulated by the addition of 1 μg/ml poly (I:C) to the culture medium. Levels of supernatant IL-6 **(A)** and expression of CD40/CD80/CD86 **(B)** were quantified 18 h later. Data are representative of two independent experiments ± s.e.m. of triplicate samples. ns, not significant (unpaired t test).
