# Supplementary material for: RNA:DNA hybrids are a novel molecular pattern sensed by TLR9
Source: EMBO J. 2014 Feb 21;33(6):542–58. doi: 10.1002/embj.201386117 (PMC3989650; doi:10.1002/embj.201386117)
Supplement: Supplementary file 6 [file embj0033-0542-sd6.pdf]

Figure S5

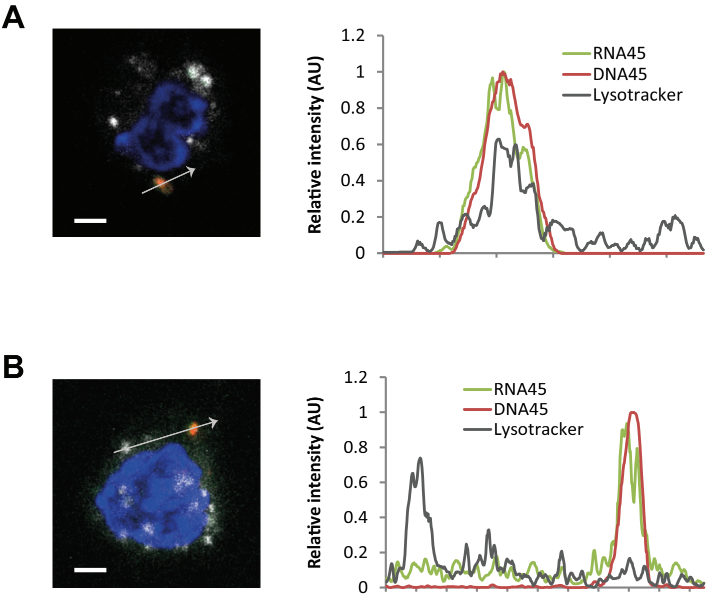
A
RNA45
DNA45
Lysotracker
Relative intensity (AU)
0
0.2
0.4
0.6
0.8
1
1.2
B
RNA45
DNA45
Lysotracker
Relative intensity (AU)
0
0.2
0.4
0.6
0.8
1
1.2

Figure S5. Fluorescently labelled R:D45 colocalises with LysoTracker, a marker of the acidified endolysosomal compartment

(A) A confocal microscope z-section of a FLDC transfected with R:D45 cell demonstrating colocalisation of fluorescently labelled hybrid with LysoTracker. Fluorescence intensity profiles of Cy3, Cy5 and LysoTracker plotted against distance, through region denoted by arrow. **(B)** Not all R:D45 foci colocalise with endolysosomes. Fluorescence intensity measurements through region denoted by arrow demonstrating an endolysosome without hybrid and a RD45 foci without endolysosomal co-staining.
