# Supplementary material for: RNA:DNA hybrids are a novel molecular pattern sensed by TLR9
Source: EMBO J. 2014 Feb 21;33(6):542–58. doi: 10.1002/embj.201386117 (PMC3989650; doi:10.1002/embj.201386117)
Supplement: Supplementary file 7 [file embj0033-0542-sd7.pdf]

Figure S6

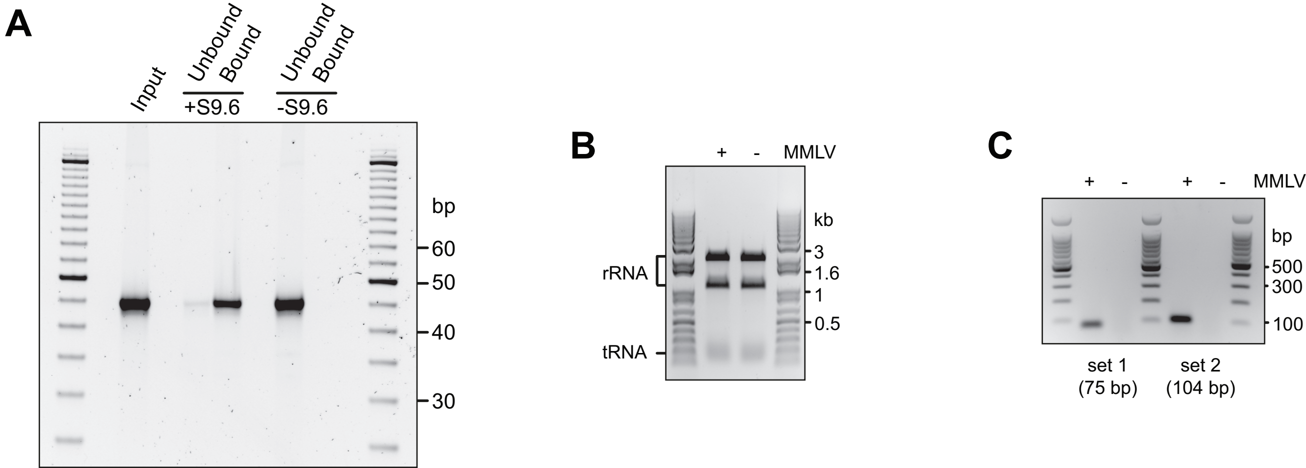
A
Input
Unbound
Bound
+S9.6
Unbound
Bound
-S9.6
bp
60
50
40
30
B
+
-
MMLV
kb
3
1.6
1
0.5
rRNA
tRNA
C
+
-
+
-
MMLV
bp
500
300
100
set 1
(75 bp)
set 2
(104 bp)

Figure S6. S9.6 pulls down synthetic and viral RNA:DNA hybrids

(**A**) S9.6 efficiently immunoprecipitates a synthetic RNA:DNA hybrid. Nearly all of 10 pmol R:D45 hybrid input is pulled down by the S9.6 antibody, whereas all hybrid is present in the unbound fraction when S9.6 is omitted in a 'beads only' control. (**B**) Total nucleic acids were isolated from B3T3 cells infected with MMLV (+) and non-infected (-), and their integrity tested by agarose gel electrophoresis showing intact rRNA and tRNA. (**C**) Both MMLV PCR primer sets specifically detect MMLV in the cytoplasmic nucleic acid fraction from infected and but not from uninfected cells
