# Supplementary material for: RNA:DNA hybrids are a novel molecular pattern sensed by TLR9
Source: EMBO J. 2014 Feb 21;33(6):542–58. doi: 10.1002/embj.201386117 (PMC3989650; doi:10.1002/embj.201386117)
Supplement: Supplementary file 8 [file embj0033-0542-sd8.pdf]

Figure S7

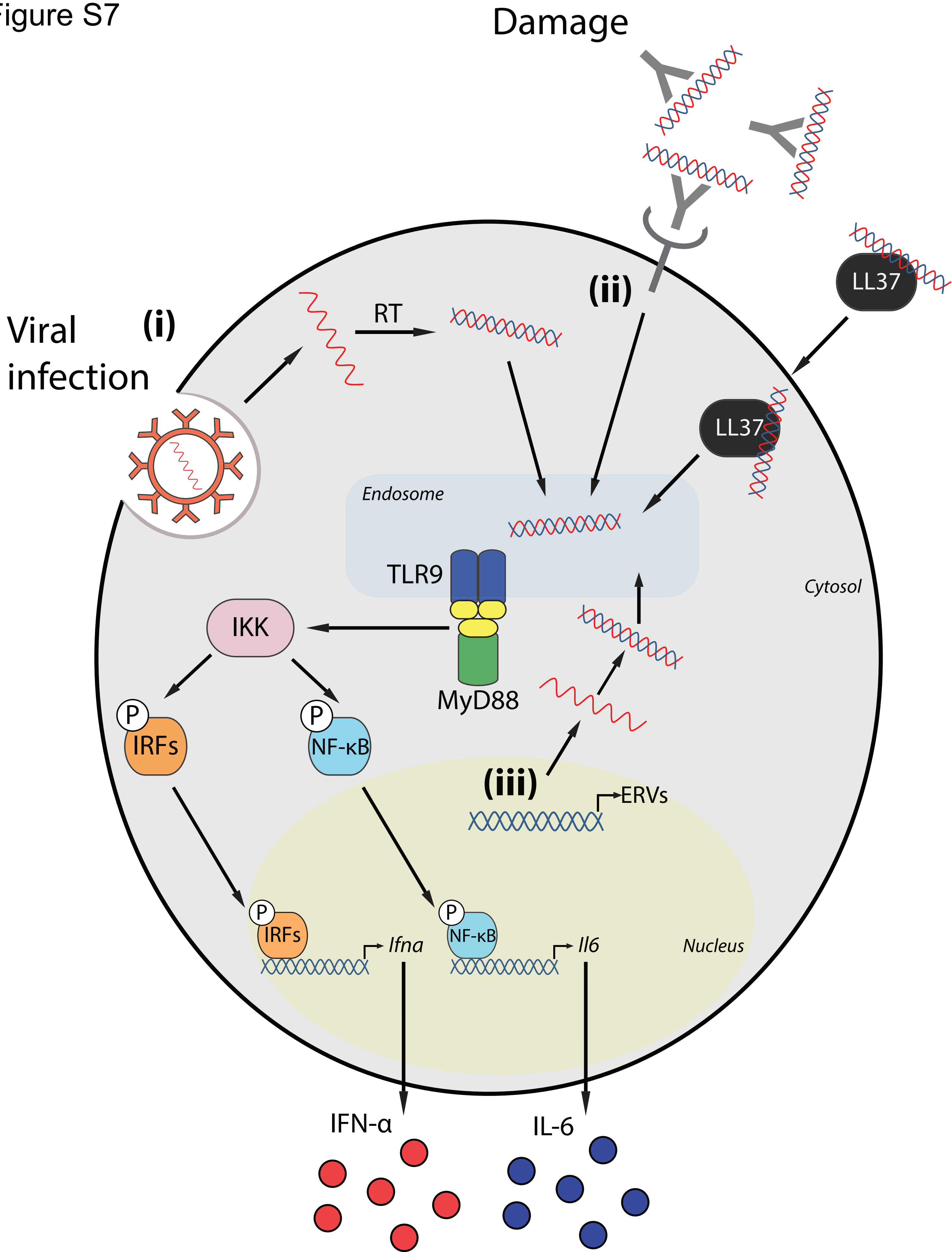
Damage
Viral
infection
(i)
RT
(ii)
LL37
LL37
Endosome
TLR9
Cytosol
IKK
MyD88
P
IRFs
P
NF-κB
(iii)
ERVs
P
IRFs
Ifna
P
NF-κB
Il6
Nucleus
IFN-α
IL-6

Figure S7. Model of the detection of RNA:DNA hybrids by dendritic cells

In pDCs, downstream signalling results in the phosphorylation and relocalisation into the nucleus of IFN regulatory factors (IRFs), such as IRF7 which is constitutively expressed in pDCs. This stimulates transcription of type I IFN genes and the subsequent secretion of large quantities of IFN-α. Downstream signalling from the RNA:DNA hybrid-TLR9-MyD88 complex in cDCs instead activates the NK-κB pathway and the induction of expression of genes encoding pro-inflammatory cytokines, including IL-6. Immunostimulatory RNA:DNA hybrids may arise from many different sources. For example, exogenous RNA:DNA hybrids generated by microbial infection **(i)** or as 'self'-nucleic acids released from damaged cells **(ii)** could enter the cell by direct endocytosis or coupling to circulating anti-nucleic acid antibodies or antimicrobial peptides, such as LL37. Once internalised, the hybrids directly, or indirectly (e.g. via autophagy) enter the endolysosomal compartment where they encounter and activate TLR9, leading to the recruitment and binding of the adaptor protein MyD88. Alternatively, an endogenous source of RNA:DNA hybrids could arise from the transcriptional activation of endogenous retroviruses (ERVs) **(iii)**, which may be transported from the cytosol to endosomes.
