# Supplementary material for: RNA:DNA hybrids are a novel molecular pattern sensed by TLR9
Source: EMBO J. 2014 Feb 21;33(6):542–58. doi: 10.1002/embj.201386117 (PMC3989650; doi:10.1002/embj.201386117)
Supplement: Supplementary file 9 [file embj0033-0542-sd9.pdf]

Table S1. Oligonucleotide sequences

Name	Sequence (5′ - 3′)	Length
**ssRNA60**	GUGUGUGUGUGUGUGUGUGUGUGUGUGUGUGUGUGUGUGUGUGUGUGUGUGUGUGUGUGU	60
**ssDNA60**	ACACACACACACACACACACACACACACACACACACACACACACACACACACACACACAC	60
**Cy3-ssRNA60**	Cy3-GUGUGUGUGUGUGUGUGUGUGUGUGUGUGUGUGUGUGUGUGUGUGUGUGUGUGUGUGUGU	60
**Cy5-ssDNA60**	Cy5-ACACACACACACACACACACACACACACACACACACACACACACACACACACACACACAC	60
**ssRNA45**	UAUCAUACCCAUUCGUCCCUCGAUCUUGCUAAGCGUCAAUUAGGA	45
**ssDNA45**	TCCTAATTGACGCTTAGCAAGATCGAGGGACGAATGGGTATGATA	45
**Cy3-ssRNA45**	Cy3-UAUCAUACCCAUUCGUCCCUCGAUCUUGCUAAGCGUCAAUUAGGA	45
**Cy5-ssRNA45**	Cy5-UAUCAUACCCAUUCGUCCCUCGAUCUUGCUAAGCGUCAAUUAGGA	45
**Cy3-ssDNA45**	Cy3-TCCTAATTGACGCTTAGCAAGATCGAGGGACGAATGGGTATGATA	45
**MMLV-F1**	GGGTGCTCCTACTCCCCTTA	20
**MMLV-R1**	TCTCATATGGGGTGAGGCCA	20
**MMLV-F2**	CAGGCCTTGCTTTTGGACAC	20
**MMLV-R2**	CAGTTGTGTTGCAGCCCTTC	20
